# Systematic analysis of specificities and flanking sequence preferences of bacterial DNA-(cytosine C5)-methyltransferases reveals mechanisms of enzyme- and sequence-specific DNA readout

**DOI:** 10.1093/nar/gkaf126

**Published:** 2025-03-04

**Authors:** Greta Sogl, Sabrina Pilling, Lukas F J Fischer, Jan Ludwig, Nahom Mihretu, Pavel Bashtrykov, Albert Jeltsch

**Affiliations:** Department of Biochemistry, Institute of Biochemistry and Technical Biochemistry, University of Stuttgart, 70569 Stuttgart, Germany; Department of Biochemistry, Institute of Biochemistry and Technical Biochemistry, University of Stuttgart, 70569 Stuttgart, Germany; Department of Biochemistry, Institute of Biochemistry and Technical Biochemistry, University of Stuttgart, 70569 Stuttgart, Germany; Department of Biochemistry, Institute of Biochemistry and Technical Biochemistry, University of Stuttgart, 70569 Stuttgart, Germany; Department of Biochemistry, Institute of Biochemistry and Technical Biochemistry, University of Stuttgart, 70569 Stuttgart, Germany; Department of Biochemistry, Institute of Biochemistry and Technical Biochemistry, University of Stuttgart, 70569 Stuttgart, Germany; Department of Biochemistry, Institute of Biochemistry and Technical Biochemistry, University of Stuttgart, 70569 Stuttgart, Germany

## Abstract

DNA-(cytosine C5)-methyltransferases (MTases) represent a large group of evolutionary related enzymes with specific DNA interaction. We systematically investigated the specificity and flanking sequence preferences of six bacterial enzymes of this class and many MTase mutants. We observed high (>1000-fold) target sequence specificity reflecting strong evolutionary pressure against unspecific DNA methylation. Strong flanking sequence preferences (∼100-fold) were observed which changed for methylation of near-cognate sites suggesting that the DNA structures in the transition states of the methylation of these sites differ. Mutation of amino acids involved in DNA contacts led to local changes of specificity and flanking sequence preferences, but also global effects indicating that larger conformational changes occur upon transition state formation. Based on these findings, we conclude that the transition state of the DNA methylation reaction precedes the covalent enzyme–DNA complex conformations with flipped target base that are resolved in structural studies. Moreover, our data suggest that alternative catalytically active conformations exist whose occupancy is modulated by enzyme–DNA contacts. Sequence dependent DNA shape analyses suggest that MTase flanking sequence preferences are caused by flanking sequence dependent modulation of the DNA conformation. Likely, many of these findings are transferable to other DNA MTases and DNA interacting proteins.

## Graphical Abstract

**Graphical Abstract:**
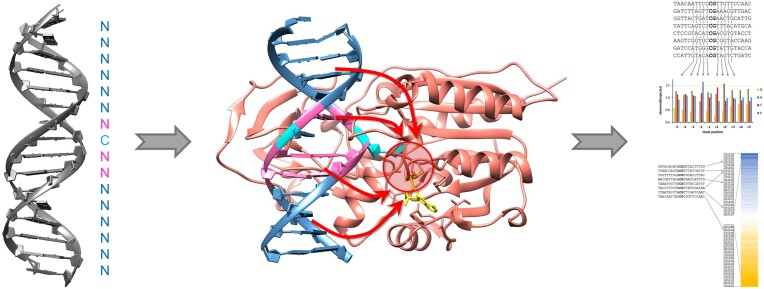

## Introduction

DNA methylation in prokaryotes plays important roles in DNA repair, restriction/modification systems, the control of DNA replication, and the regulation of gene expression [1, 2]. It occurs at the adenine-N6, cytosine-C5, and cytosine-N4 positions and is introduced by distinct classes of DNA methyltransferases (MTases) specifically within short recognition sequences, typically 2–6 bp in length [3]. Bacterial DNA MTases were shown to possess a wide variety of sequence specificities [4] and they represent a paradigm of a large, diverse group of enzymes interacting with different DNA sequences in a highly specific manner. These enzymes are the outcome of divergent evolution [5] as documented by their structural and amino acid sequence similarities [6, 7]. The specific activity of DNA MTases is critically connected to their biological roles, e.g. in phage protection, DNA damage repair, control of cell cycle and gene expression, because aberrant methylation would disrupt the DNA interaction of a myriad of other cellular proteins creating a strong selection pressure for specificity in DNA methylation. However, despite many years of research on DNA MTases, the specificities of these enzymes are insufficiently characterized and quantitative data on the sequence specificity of bacterial MTases is still lacking. Of note, the sequence specificity of this class of enzymes has two dimensions, the first is dealing with the specific methylation of the target motif characteristic for each enzyme. The second dimension refers to the impact of DNA sequences outside of the target motif on the methylation activity which we call flanking sequence preferences here. Both of these effects are mediated by direct contact of the enzyme to the edges of the DNA bases mainly in the major groove of the DNA and sequence specific effects on the structure and dynamics properties of the DNA, which determine the match of a DNA substrate with a particular sequence into the DNA-binding site of the corresponding enzyme [8, 9].

In this work, we systematically investigated the DNA methylation specificity and the influence of the flanking sequences surrounding the target sites on the methylation activities for a panel of six representative bacterial DNA-(cytosine C5)-MTases, *viz*. M.SssI (CG), M.HhaI (GCGC), M.HaeIII (GGCC), M.HpaII (CCGG), M.MspI (CCGG), and M.AluI (AGCT) (target sequences are indicated in brackets with the target cytosine underlined). Except M.SssI, these enzymes are parts of bacterial restriction/modification systems. M.SssI had been discovered and its specificity to methylate CG sequences determined in the 1990s [10, 11]. Due to the fact that its CpG specificity mirrors eukaryotic DNA methylation patterns, the enzyme has found applications in research and biotechnology [12–19]. However, there are no systematic studies investigating the activity of this enzyme at CpG and non-CpG sites in different flanking sequence contexts. M.HhaI was discovered and its GCGC specificity determined in the 1970s [20, 21]. This enzyme represents the best studied bacterial DNA-(cytosine C5)-MTase and the first protein–DNA complex structure for this class of enzymes was solved for M.HhaI leading to the breakthrough discovery of base flipping [22, 23]. Nuclear Magnetic Resonance experiments revealed a large structural plasticity of the protein upon addition of the coenzyme AdoMet, including allosteric structural and dynamic changes at sites in large distances from the AdoMet binding site [24]. M.HaeIII had been discovered and shown to methylate GGCC sequences in 1977 [25] and cloned in 1988 [26]. It represents the second MTase–DNA complex structure that was resolved [27], revealing for the first-time massive rearrangements of the DNA structure after base flipping, in which the orphaned G forms a bp with the adjacent C in the target motif and the corresponding G becomes orphaned. Among the additional enzymes studied here, M.HpaII and M.MspI were chosen as examples of enzymes recognizing the same DNA sequence but methylating different cytosine residues (CCGG and CCGG, respectively). M.AluI (ACGT) was selected as an example of an enzyme with a more AT-rich target sequence.

The reason for our knowledge gap in the quantitative description of the specificity of enzyme–DNA interaction is that it requires the investigation of enzyme activity with a very large number of DNA substrates with different sequences. Assuming an enzyme has a 4 bp recognition sequence and three neighboring bases on either side have relevant effects on activity, methylation rates of 4^10^ = 1.048.576 different DNA substrates would have to be studied, which was not possible with conventional technologies. Hence, experimental studies of MTase specificity often employed qualitative *in vitro* or *in vivo* assays [28–30]. In case of M.HaeIII, cellular methylation efficiencies were studied on plasmids revealing some promiscuous activity of this enzyme [29], which later could be modulated by evolutionary enzyme design leading to the development of M.HaeIII mutants with altered specificity [31]. Quantitative kinetic studies investigating the specificity of DNA MTases typically were based on the kinetic analysis of a relatively small number of different substrates [32]. Only in few cases, biochemical experiments were systematically conducted to determine the level of preference for the canonical target sequence over near-cognate sites (sites differing by one bp from the cognate site). For example, in case of the adenine-N6 MTase EcoDam, several synthetic DNA substrates were used and 100- to 1000-fold preferences were observed for the methylation of GATC sites when compared with all existing near-cognate sites [33, 34]. A systematic study investigated DNA recognition of M.HhaI at the first and third position of the recognition sequence and revealed high specificity of approximately 100-fold in each case which was even more elevated in the context of a H127A/T132A double mutant affecting DNA backbone contacts of the enzyme [35]. However, for technical reasons, all these studies were conducted in only one (or few) flanking DNA sequence context and, hence, they only covered a small fraction on the potential DNA sequence space these enzymes are facing in cells.

In this context, it is of relevance that previous work with mammalian DNA methyltransferases (DNMTs) has shown unexpected strong effects of the sequences flanking the actual recognition site on the enzymatic activity [36]. These preferences arise from the fact that the DNA interface of MTase–DNA complexes typically covers around 10–12 bp, exceeding the 2–6 bp of the core recognition sequence. Therefore, MTases also interact with the DNA outside of the core recognition motif and sequence dependent changes of the DNA structure and its dynamics can directly affect DNA binding and the frequency of transition state formation. In many cases, methylation rates of target sites differed up to 100-fold in the context of different flanking sequences and the accuracy of the target sequence recognition was also flanking sequence dependent [37–43]. These findings illustrate that the interaction of MTases with the core recognition sequence and flanking sequences are connected and they have the ability to mutually affect each other, indicating that methylation of cognate and noncognate substrates should ideally be studied in a variety of different sequence contexts. Here, we have applied a recently developed Deep Enzymology method to conduct this type of experiment with a panel six representative bacterial DNA-(cytosine C5)-MTases and 24 mutants of M.HhaI and M.HaeIII selected to specifically disrupt individual enzyme–DNA contacts. In Deep Enzymology experiments, DNA substrates are used, which contain the target cytosine in a randomized sequence context. After incubation of the library of substrate molecules with the MTase and methylation of some of the substrate molecules, unmethylated cytosine residues are converted by bisulfite to U, which is later amplified and sequenced as T allowing to distinguish methylated and unmethylated cytosine residues. The library of product molecules is then analyzed by next-generation sequencing (NGS) allowing for each individual molecule to determine its methylation state and DNA sequence. Based on this, detailed information can be derived regarding the influence of the sequence in the randomized part of the substrate on enzyme activity. To generate robust data with high dynamic range for the detection of effects, several methylation experiments were conducted for each enzyme and combined for data analysis, in total 196 experiments comprising >1300 000 individual sequence reads (Supplementary Table S1). This allowed us to determine the recognition specificity and flanking sequence preferences (and their mutual connection) quantitatively and with high resolution providing interesting and novel insights into the reaction mechanism of DNA-(cytosine C5)-MTases. Considering these DNA MTases an exemplary, well-studied group of evolutionary related enzymes that specifically interact with DNA, many of our results are likely to be transferable to other DNA interacting enzymes and proteins.

## Materials and methods

### DNA MTases cloning, expression, and purification

The synthetic genes for M.HhaI wildtype (WT), M.HaeIII WT, and M.HpaII were obtained from Integrated DNA Technologies (IDT) and cloned into the TOPO-TA vector pSC-A from the StrataClone polymerase chain reaction (PCR) Cloning Kit (Agilent Technologies). Afterwards, Gibson Assembly was performed to clone them into a pBAD-amp expression vector with an N-terminal His_6_-tag. Site-directed mutagenesis was performed [44] to introduce the mutations K89A, R97A, S126A, K162A, R209A, T226A, R228A, K234A, Q237A, R240A, Y242A, T250A, S252A, S294A, S296A, and Q297A in M.HhaI as well as S79A, R81A, R87A, S219A, S224A, R225A, R243A, and Q244A in M.HaeIII. The cloning and mutagenesis were verified by Sanger sequencing (Microsynth Seqlab GmbH). The M.SssI gene was taken from [45].

M.SssI, M.HhaI WT and mutants, M.HaeIII WT and mutants, and M.HpaII were overexpressed using NEBExpress^®^ competent *Escherichia coli* (High Efficiency) cells after the transformation of the respective plasmids. Pre-cultures of the cells were grown in Lysogeny Broth (LB) medium supplemented with 1% D-(+)-glucose at 37°C for 16 h. For the main cultures, the pre-cultures were diluted 200-fold and subsequently supplemented with 0.1% D-(+)-glucose. After an OD_600_ of 0.6–0.8 was reached, the protein expression was induced by adding 1% L-(+)-arabinose and the proteins were expressed at 37°C for 2 h with horizontal shaking.

For protein purification, Ni-NTA/His affinity chromatography was used. All steps were carried out at 4–6°C. For the cell lysis, the harvested cells were each resuspended in 20 ml washing buffer (20 mM Tris, 1 M NaCl, 10 mM imidazole, 0.1 mM dithiothreitol (DTT), pH 7.5) supplemented with 40 μl phenylmethylsulfonyl fluoride (PMSF) and 100 μl Protease Inhibitor Cocktail (PIC; 300 μM AEBSF-HCl, 3 μM pepstatin A, 0.12 μM aprotinin, 15 μM bestatin, 4.5 μM E-64, 6.4 μM leupeptin) and sonicated 15 times with 15 s impulse (four cycles, 30% power) and 45 s off-time per repeat (Q120 Sonicator, Active Motif). The lysed cells were then centrifuged (47 850 × *g*, 1 h) and the lysate was added to 500 μl of Ni-NTA agarose beads (QIAGEN) previously equilibrated to the washing buffer with PMSF and PIC. This mixture was rotated for 1 h to bind the His-tagged proteins to the beads. Afterwards, the beads were loaded on the column and washed with 150 ml washing buffer. Elution was carried out with elution buffer (20 mM Tris, 200 mM NaCl, 200 mM imidazole, 0.1 mM DTT, 10% glycerol, pH 7.5) in 12 fractions of roughly 150 μl. The absorption of the single fractions was measured at 280 nm and protein concentrations were calculated using the protein extinction coefficients. Afterwards, the 4–5 fractions with the highest protein concentrations were combined for dialysis. Dialysis I was performed for 2 h in 1 l dialysis I buffer [20 mM Tris, 200 mM NaCl, 1 mM ethylenediaminetetraacetic acid (EDTA), 0.1 mM DTT, 10% glycerol, pH 7.5] followed by dialysis II in 0.5 l dialysis II buffer (20 mM Tris, 400 mM NaCl, 1 mM EDTA, 0.1 mM DTT, 70% glycerol, pH 7.5) for 16 h. Aliquots of the purified proteins were flash-frozen in liquid nitrogen and stored at −80°C. The purity and concentrations of the proteins were determined using sodium dodecyl sulphate–polyacrylamide gel electrophoresis (10% acrylamide) and subsequent Coomassie BB staining for M.SssI, M.HaeIII WT and mutants, and M.HpaII. For M.HhaI WT and mutants, the concentrations of the individual preparations were equalized based on the band intensities in semi-dry western Blots with an anti-His antibody (QIAGEN, ID: 34 670). M.AluI and M.MspI were purchased from New England Biolabs.

### Radioactive DNA methylation assay

For the methylation activity measurement of the bacterial DNMTs, a biotin–avidin microplate assay was basically performed as described previously [46]. As a substrate, an unmethylated biotinylated double stranded 30mer oligodeoxynucleotide containing the respective methylation target site of the investigated proteins was used in a concentration of 0.122 μM. The methylation reactions contained 0.1 μM radioactively labelled [methyl-^3^H]-S-adenosyl-L-methionine (Perkin Elmer) and, depending on the enzyme, different methylation buffers were used: for M.SssI NEBuffer™2 (New England Biolabs), for M.HhaI and M.HpaII rCutSmart (New England Biolabs), for M.HaeIII 1× M.HaeIII methylation buffer (New England Biolabs), for M.AluI 1× AluI Methylase Reaction Buffer (New England Biolabs), and for M.MspI 1× MspI Methylase Reaction Buffer (New England Biolabs). Different enzyme concentrations and time points were used depending on the MTase as indicated in the text. The reactions were started with the substrate and conducted at 37°C. At appropriate time points, aliquots of 2 μl were withdrawn and added to one well of an avidin-coated microtiter plate containing 40 μl of stop solution [1.25 mM unlabeled AdoMet (Sigma) dissolved in 0.5 M NaCl, 1× PBST: 137 mM NaCl, 2.7 mM KCl, 10 mM Na_2_HPO_4_, 1.8 mM KH_2_PO_4_, 0.05% Tween^®^ 20, pH 7.4] to quench the reaction. After binding the biotinylated substrates to the avidin by horizontal shaking (20 min), the wells were washed with washing buffer (0.5 M NaCl, 1× PBST) to remove unreacted AdoMet. Afterwards, the bound substrates were digested using the nonspecific *Serratia marcescens* endonuclease (700 U, 1 h). The radioactivity was quantified by liquid scintillation counting after adding the mixture in 3 ml scintillation solution (Rotiszint^®^ eco plus solution) using the Liquid Scintillator Counter Hidex 300SL. Subsequently, the counts per minute (CPM) were plotted against the time and a least square fit of the data to Equation 1, a monoexponential reaction progress curve with *A* representing the maximal CPM value and *k* the reaction rate constant, was performed. For the determination of the initial methylation activity, the initial reaction velocity was calculated. Therefore, Equation 1 was differentiated to derive the reaction rate (*v*) (Equation 2), indicating at *t*= 0 that the product of *A* and *k* represents the initial reaction rate (Equation 3). All reactions were carried out at least in duplicates.


(1) \begin{eqnarray*}{\rm CPM}\left( t \right) = A \cdot \left( {1 - {{e}^{ - k \cdot t}}} \right)\end{eqnarray*}

(2) \begin{eqnarray*}v = A \cdot k \cdot {{e}^{ - k \cdot t}}\end{eqnarray*}

(3) \begin{eqnarray*}v\left( 0 \right) = A \cdot k\end{eqnarray*}

### Methylation assay and generation of NGS library

For the analysis of the enzyme specificity, substrates were synthesized containing the target C in a CN dinucleotide with 10 randomized base pairs at the 5′- and 11 at the 3′-end (CNrand). Therefore, two single-stranded DNA oligonucleotides with a CpH site (H denoting A, T, or C) (CHrand) or a CpG site (CGrand) were purchased from IDT (GAG TGT GAC TAG GCT CTC ACT GCC NNN NNN NNN NCHN NNN NNN NNN GAG AGG AGA CCT AGT GAG AAG or GAG TGT GAC TAG GCT CTC ACT GCC NNN NNN NNN NCGN NNN NNN NNN GAG AGG AGA CCT AGT GAG AAG; N denoting A, G, T, or C). The second strand was synthesized using a primer extension reaction and the obtained CpH and CpG substrates were combined in a 3:1 ratio (CpH:CpG) resulting in the CNrand substrate. This substrate was used to determine methylation at noncognate sites, and, if methylation levels were sufficient, to derive the flanking sequence preferences for these events. For the flanking sequence preference analysis at the canonical site, the substrates were synthesized the same way using similar oligonucleotides with the target motif of the respective MTase embedded in nine randomized nucleotides on both sides.

In each case, 100 ng of the substrates were used for methylation reactions using 1 μM *S-*adenosyl-L-methionine (Sigma) in the same methylation reaction buffers that were used for the biotin–avidin microplate methylation assays. The reaction mixtures were incubated at 37°C and afterwards snap-frozen in liquid nitrogen to stop the methylation reaction at specific time points that were chosen based on the determined methylation activities. After a Proteinase K digestion (New England Biolabs, 80 U in reaction, 1 h, 50°C), the samples were purified using the NucleoSpin^®^ Gel and PCR Clean-up Kit (Macherey-Nagel). The DNA was then digested with BsaI-HFv2 (New England Biolabs) and the upper and lower DNA strands were connected by ligating a stem-loop oligodeoxynucleotide (pGAGAAGGGATGTGGATACACATCCCT) to the cut end using T4 DNA ligase (New England Biolabs). To differentiate methylated and unmethylated cytosine, the DNA was bisulfite converted with the EZ DNA Methylation-Lightning™ Kit (Zymo Research). Afterwards, libraries for Illumina NGS were generated using a two-step PCR approach with varying primers for the introduction of sample specific barcode and index combinations to distinguish the samples from each other [47]. Illumina NGS was performed by Novogene Co., Ltd.

### Bioinformatic analysis

The analysis of the NGS data was performed using a local Galaxy server [48] basically as described previously [38,42]. In brief, the fastqsanger files received from the sequencing company were processed with the Trim tool to 128 nucleotides. Afterwards, the reads were filtered for a quality score of at least 20 with FASTQ. Next, duplicate reads were removed and using the sequence information of both strands, the original flanking sequence before bisulfite conversion was determined using a self-written Python script. Sequences with mismatches in this step were discarded. Then, the methylation state of all cytosines was determined considering the sequences of the upper and the lower DNA strand using a Python script. For further analysis, only the reads of the upper DNA strand were used. For the analysis of the substrate specificity, the CNrand substrate was used and methylation was analyzed in all potential sequence motifs: M.SssI: 5′-CN-3′, M.HhaI/M.HpaII: 5′-NCNN-3′, M.HaeIII/M.AluI: 5′-NNCN-3′, and M.MspI: 5′CNNN3’. Then, the average methylation levels of the respective cognate sites as well as the methylation levels of all near-cognate sites (which differ from the target sequence by 1 nucleotide) were determined. Moreover, average methylation levels in all methylated and unmethylated 5′-NNXNN-3′ and 5′-NNNXNNN-3′ motifs (X representing the cognate motif) were determined and compared to detect combinatorial sequence effects. The methylation levels of all 256 5′-NNXNN-3′ sequences were also used to create Pearson *r*-values for correlation analysis of individual data sets.

For analysis of flanking sequence preferences, the occurrence of each base at each position in the flanking sites in methylated sequence reads was determined and compared with the overall distribution of nucleotides at the corresponding position. This was followed by calculation of the position specific enrichment or depletion of each base in the flanking sites of methylated reads which is reported in observed/expected (o/e). This describes the preference of the corresponding enzyme to methylate target sites in the respective flanking sequence context. The overall effect at site i (E_i_) is calculated with equation 4, where A_i_, G_i_, T_i_ and C_i_ represent the corresponding o/e values for A, G, T, or C:


(4) \begin{eqnarray*} {E}_{i} &=& \sqrt {{\left( {A}_{i} - 1 \right)}^{2} + { \left( {{{T}_i} - 1} \right)}^{2}+{ \left( {{{T}_i} - 1} \right)}^{2} + {\left( {C}_{i} - 1 \right)}^{2}} \end{eqnarray*}

### DNA shape analyses

DNA shape features (Supplementary Fig. S1) include 6 bp parameters describing translations (Shear, Stretch, and Stagger), and rotations [Buckle, Propeller Twist (ProT), and Opening] of the two bases within a bp. Moreover, 6 bp step parameters describe the translations (Shift, Slide, and Rise), and rotations [Tilt, Roll, and Helix Twist (HelT)] between two adjacent base pairs. Finally, two minor groove features describe minor groove width (MGW) and minor groove electrostatic potential (EP). EP is affected by the positively polarized G amino group in the minor groove which partially neutralizes the negative DNA potential. Of note, EP and MGW are correlated in narrow minor-groove regions, but MGW is not a general approximation for EP [49–51].

**Figure 1.**
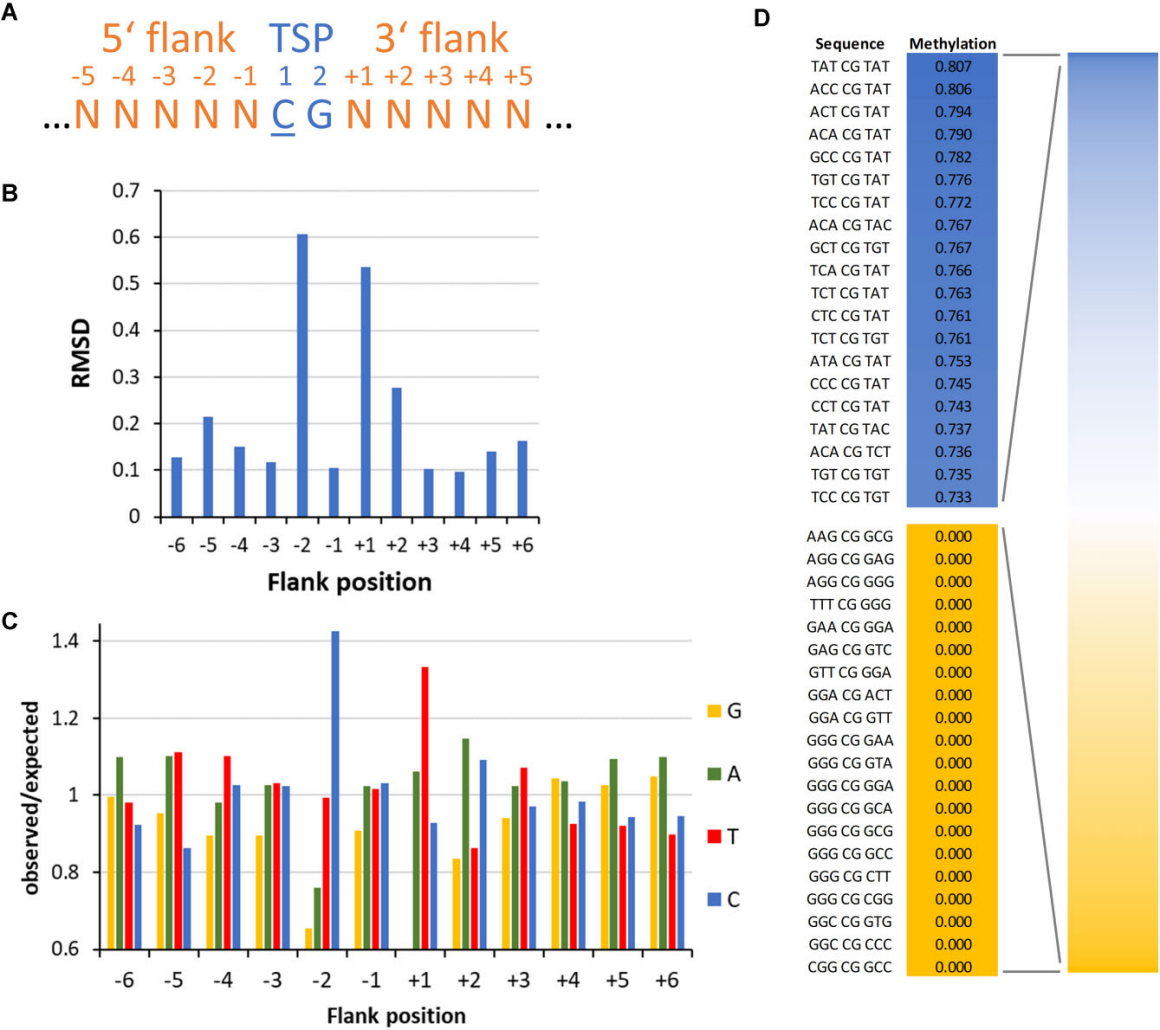
Flanking sequence preferences of M.SssI in CpG methylation determined with a substrate library containing a CG site in randomized sequence context. (**A**) Scheme of the sequence numbering and designations used in this work. TSP, target site position. (**B**) Magnitude of the flanking sequence preferences in CpG methylation at the −6 to +6 flank positions. Enrichment and depletion of bases at the positions in methylated substrate molecules were determined and the position-specific effect size plotted. Also see Supplementary Fig. S4B. (**C**) Enrichment and depletion of bases at the positions in methylated substrate molecules were determined and plotted as o/e frequencies. (**D**) Average methylation levels of NNNCGNNN bins.

DNA shape features were predicted using the Deep DNAshape server (https://deepdnashape.usc.edu/) [51, 52] for all N_6_XN_6_ sequences (where X denotes the MTase target sequence) using Deep DNAshape layer 4. Predictions were made for all enzymes studied here using the appropriate target sequences. These data were used to extract averaged N_4_XN_4_ values for each parameter. Relative methylation activities (Act) of all enzymes were calculated for all 65,536 N_4_XN_4_ sequences on the basis of the position specific enrichment or depletion of nucleotides in the pool of methylated product molecules (shown in Figs 1C, 4B, 7B, and 8A–C) using Equation 5.


(5) \begin{eqnarray*}\begin{array}{@{}l@{}} {\mathrm{Act }}\left( {{{{\mathrm{N}}}_{{\mathrm{ - 6}}}}{{{\mathrm{N}}}_{{\mathrm{ - 5}}}}{{{\mathrm{N}}}_{{\mathrm{ - 4}}}}{{{\mathrm{N}}}_{{\mathrm{ - 4}}}}{{{\mathrm{N}}}_{{\mathrm{ - 3}}}}{{{\mathrm{N}}}_{{\mathrm{ - 2}}}}{{{\mathrm{N}}}_{{\mathrm{ - 1}}}}{{{\mathrm{X}}}_{\mathrm{1}}}{{{\mathrm{X}}}_{\mathrm{2}}}{{{\mathrm{X}}}_{\mathrm{3}}}{{{\mathrm{X}}}_{\mathrm{4}}}{{{\mathrm{N}}}_{{\mathrm{ + 1}}}}{{{\mathrm{N}}}_{{\mathrm{ + 2}}}}{{{\mathrm{N}}}_{{\mathrm{ + 3}}}}{{{\mathrm{N}}}_{{\mathrm{ + 4}}}}{{{\mathrm{N}}}_{{\mathrm{ + 5}}}}{{{\mathrm{N}}}_{{\mathrm{ + 6}}}}} \right){\mathrm{ = }}\\ {{{\mathrm{f}}}_{{\mathrm{ - 6}}}}\left( {{{{\mathrm{N}}}_{{\mathrm{ - 6}}}}} \right) \times {{{\mathrm{f}}}_{{\mathrm{ - 5}}}}\left( {{{{\mathrm{N}}}_{{\mathrm{ - 5}}}}} \right) \times {{{\mathrm{f}}}_{{\mathrm{ - 4}}}}\left( {{{{\mathrm{N}}}_{{\mathrm{ - 4}}}}} \right) \times {{{\mathrm{f}}}_{{\mathrm{ - 3}}}}\left( {{{{\mathrm{N}}}_{{\mathrm{ - 3}}}}} \right) \times {{{\mathrm{f}}}_{{\mathrm{ - 2}}}}\left( {{{{\mathrm{N}}}_{{\mathrm{ - 2}}}}} \right) \times {{{\mathrm{f}}}_{{\mathrm{ - 1}}}}\left( {{{{\mathrm{N}}}_{{\mathrm{ - 1}}}}} \right) \times \\ {{{\mathrm{f}}}_{{\mathrm{ + 1}}}}\left( {{{{\mathrm{N}}}_{{\mathrm{ + 1}}}}} \right) \times {{{\mathrm{f}}}_{{\mathrm{ + 2}}}}\left( {{{{\mathrm{N}}}_{{\mathrm{ + 2}}}}} \right) \times {{{\mathrm{f}}}_{{\mathrm{ + 3}}}}\left( {{{{\mathrm{N}}}_{{\mathrm{ + 3}}}}} \right) \times {{{\mathrm{f}}}_{{\mathrm{ + 4}}}}\left( {{{{\mathrm{N}}}_{{\mathrm{ + 4}}}}} \right) \times {{{\mathrm{f}}}_{{\mathrm{ + 5}}}}\left( {{{{\mathrm{N}}}_{{\mathrm{ + 5}}}}} \right) \times {{{\mathrm{f}}}_{{\mathrm{ + 6}}}}\left( {{{{\mathrm{N}}}_{{\mathrm{ + 6}}}}} \right) \end{array}\end{eqnarray*}

with:

*N_i_*: nucleotide present at flank position i

*X_i_*: nucleotide in the target sequence (in case of M.SssI only X_1_ and X_2_ were used)

*f_i_(N)*: o/e value of nucleotide N at flanking position i in the enzyme’s preference profile

The MTase flanking sequence dependent activities and DNA shape data for the N_4_XN_4_ sequences were correlated in MS Excel and the corresponding Pearson *r*-values determined. Intrinsic DNA shape parameters of the different target sequences were determined by averaging the DNA shape profiles of all 65,536 N_4_XN_4_ sequences.

### Quantification and statistical analysis

All details regarding statistical analyses are provided in the respective text, figure legends and figures, including numbers of replicates for each experiment, statistical tests used and the obtained Pearson *r*-values.

## Results and discussion

Enzymes acting on DNA with sequence-specificity include several classes of bacterial and eukaryotic DNA-(cytosine C5)-MTases [3, 53, 54]. It was the aim of this study to investigate the details of the DNA interaction specificity of bacterial DNA MTases of this class using a Deep Enzymology approach [36], focusing on their target site specificity, flanking sequence preferences and the crosstalk of these properties. In our experimental approach, DNA substrate libraries are prepared containing target cytosine residues in the corresponding MTase target motif surrounded by randomized flanking sequences. The different substrate libraries are incubated with the corresponding MTases and DNA methylation is analyzed by bisulfite conversion followed by NGS (Supplementary Fig. S2A). For each enzyme, a DNA substrate library containing the specific recognition sequence of the enzyme in the center surrounded by randomized flanking sequences was prepared and analyzed. Then, the reads were split in methylated and unmethylated ones and the occurrence of each base at each position in the flanking sites in methylated sequence reads was determined. This was compared with the overall distribution of nucleotides at the corresponding position leading to the computation of the position specific enrichment or depletion of each base at each position in the flanking sites of methylated reads. This describes the preference of the corresponding enzyme to methylate target sites in the respective flanking sequence context (Supplementary Fig. S2B). Moreover, methylation levels were averaged in NNNXNNN bins, where N stands for any nucleotide (A, G, T, or C) and X stands for the corresponding recognition motif (2 or 4 nucleotides in length). This analysis allowed a quantitative assessment of the degree of flanking sequence preferences and detection of potential complex preference patterns and complex sequence effects (Supplementary Fig. S2D). For specificity analysis, a substrate containing a CpN dinucleotide sequence (CN site) in a randomized sequence context was methylated by each enzyme. From these data, the sequence reads corresponding to the cognate recognition motif of the corresponding enzyme and all near-cognate target sites (defined as sites with one bp deviation from the cognate motif) were extracted and their averaged methylation levels (Supplementary Fig. S2C) used to determine the specificity of the enzyme for its target motif and the least and most preferred near-cognate motifs. Depending on the available sequencing depth and methylation levels, flanking sequence preference data were also derived in a similar manner for the methylation of near-cognate sites allowing to assess the connection of target site recognition and flanking sequence interaction.

### Specificity and flanking sequence preferences of the DNA methylation by M.SssI

M.SssI was expressed in *E. coli*, purified as described [55] (Supplementary Fig. S3A), and its activity validated with radioactive DNA methylation assays (Supplementary Fig. S4A). For the flanking sequence preference analysis, we first used a library of double stranded substrate DNA molecules containing one central hemimethylated CpG site in a randomized sequence context to focus the methylation activity on one DNA strand (Fig. 1A). The mixture of DNA molecules was incubated with different concentrations of M.SssI in methylation buffer and samples were taken at different time points yielding in total five experimental repeats (Supplementary Table S1). Afterward, the samples were bisulfite converted and sequenced. From the sequencing data, the average methylation levels of each of the 256 NNCGNN sequence contexts were determined. Correlation analysis revealed a high similarity between the corresponding profiles of all five repeats (Supplementary Fig. S4B) and, therefore, the data were pooled. Next, the frequency of each base at each of the −6 to +6 flanking sites was determined in the methylated reads and compared with the frequency of the same base at the corresponding site in the overall pool. Strongest flanking sequence preferences were observed at the −2 to +2 sites (Fig. 1B). M.SssI showed a preference for C at the −2 flanking site, here denoted as C(−2), and disfavor for G(−2). The overall preference at the −2 site (C > T > A > G) indicates that pyrimidines are preferred. In addition, we observed preferences for T(+1) and A(+2) or C(+2). Interestingly, the flanking sequence preferences were strong at the −2 site, but very weak at −1 although this bp is the direct neighbor of the flipped target C. Comparison of the averaged methylation levels of all 4096 NNNCGNNN bins (Fig. 1C) revealed 80.7% methylation of the best flanking site (TAT-CG-TAT), which is an example of a complex sequence effect, as it does not contain a C(−2) though this base was most preferred at the −2 site in the isolated analysis. In contrast, several NNN-CG-NNN sites showed no methylation at all. As expected, all these sequences contained several disfavored bases. The unmethylated sites had a combined sequence coverage of 847 reads indicating that their methylation level is below 0.35% (binomial distribution, p=0.05). Considering exponential rate fitting for the most the methylaiton of the most preferred sites, the flanking sequence preferences of M.SssI are >400-fold, even stronger than observed with other MTases before, *viz*. DNMT1 [38,42], DNMT3A [37,39,41], DNMT3B [39, 40], and DNMT3C [43]. However, the M.SssI flanking sequence preferences are completely different from preferences observed with these human and mouse CpG specific MTases in similar experiments, indicating that the DNA interaction of M.SssI is unique despite of its interaction with the same CpG target site.

**Figure 2.**
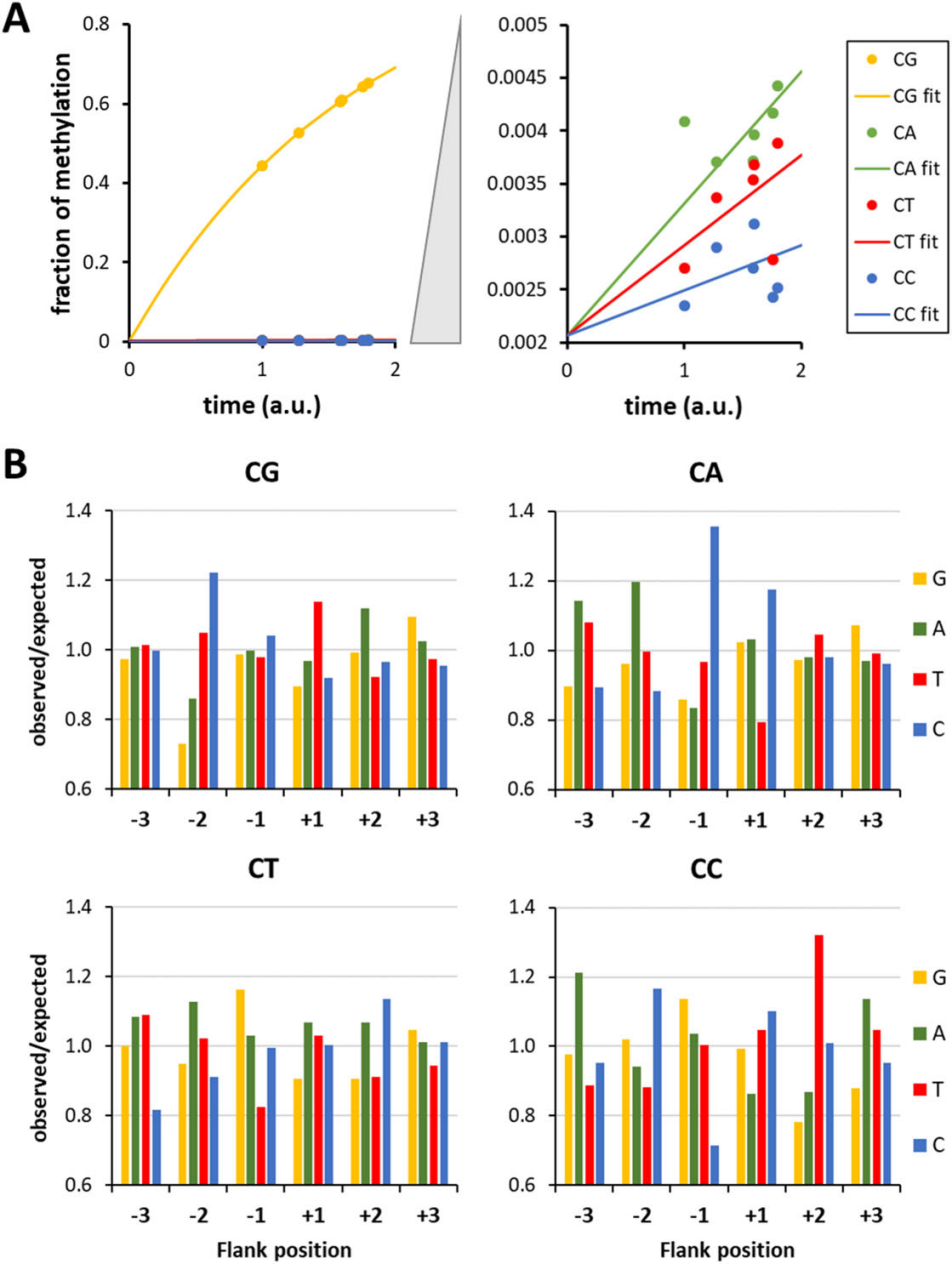
CpG specificity of M.SssI determined with substrate library containing a CN site in a randomized sequence context. (**A**) Average methylation of CpG, CpA, CpT, and CpC sites in the different experiments fitted to an exponential reaction progress curve. Also see Supplementary Fig. S4C. (**B**) Flanking sequence preferences of M.SssI in CpG and non-CpG methylation. Shown are the enrichment and depletion of bases at the −2 to +2 flank positions in substrate molecules methylated at CpG, CpA, CpT, and CpC sites. Also see Supplementary Fig. S4C.

To analyze the CpG and non-CpG methylation activity of M.SssI, a library of double stranded substrate DNA molecules containing one central CN target site in a randomized sequence context was methylated and analyzed in six independent experiments (Supplementary Table S1). We extracted the reads containing the central C in CpG, CpA, CpT, and CpC context and determined their average methylation levels. To derive apparent first order rate constants for methylation in the CpG and non-CpG context, the methylation data of the individual experiments were fitted to exponential reaction progress curves using a virtual time axis. This analysis revealed very high specificity for CpG methylation (Fig. 2A). CpA was the second-best methylation target with a methylation rate of about 450-fold below that of CpG. CpT methylation was 650-fold slower than CpG, CpC methylation was even lower. With this high level of CpG specificity, M.SssI resembles the mammalian DNMT1 enzyme [38,42], while DNMT3 enzymes showed much higher levels of non-CpG methylation in similar experiments [39–41,43].

Using the CN methylation data, we next determined the CpG methylation levels of NNCGNN bins in the six independent experiments. Correlation analysis revealed a high similarity between all profiles (Supplementary Fig. S4C). Hence, the data sets were combined and the CpG and non-CpG flanking sequence preferences were extracted focusing on the 3 bp surrounding the target site (Fig. 2B). The CpG flanking sequence preferences determined with the CN substrate were very similar to the ones determined with the CpG substrate (compare Figs 1C and 2B). The small differences can be explained by the higher overall methylation levels of the CpG sites in the CN data sets and the smaller number of available sequence reads. This analysis revealed the unexpected finding that flanking sequence preferences were completely different in all four methylation contexts, *viz*. CpG, CpA, CpT, and CpC. Of note, CpA methylation has a strong C(−1) preference and CpC a preference for T(+2) which both are completely different from the preferences at these sites in CpG methylation. Moreover, the C(−2) preference, the strongest effect in CpG methylation, is not observed in CpA and CpT methylation, which both show preferences for A(−2). Finally, in non-CpG methylation, strong flanking sequence effects were also observed that the −3 and +3 sites, where CpG methylation did not show effects. The distinct flanking sequence preferences observed in CpG, CpA, CpT and CpC methylation and the large deviations in flanking sequence preferences at positions far away from the variable CpX residue indicate that the DNA conformations in the transition states of the CpX methylation events are different. It is conceivable that different structural rearrangements of the DNA occur after base flipping in the four structural contexts reminiscent of what has been observed in DNMT1-DNA complexes obtained with DNA substrates with different flanking sequences [38,56]. Future structural and molecular dynamics simulation studies will be needed to understand the mechanistic basis on these effects.

### Specificity and flanking sequence preferences of the DNA methylation by M.HhaI

**Figure 3.**
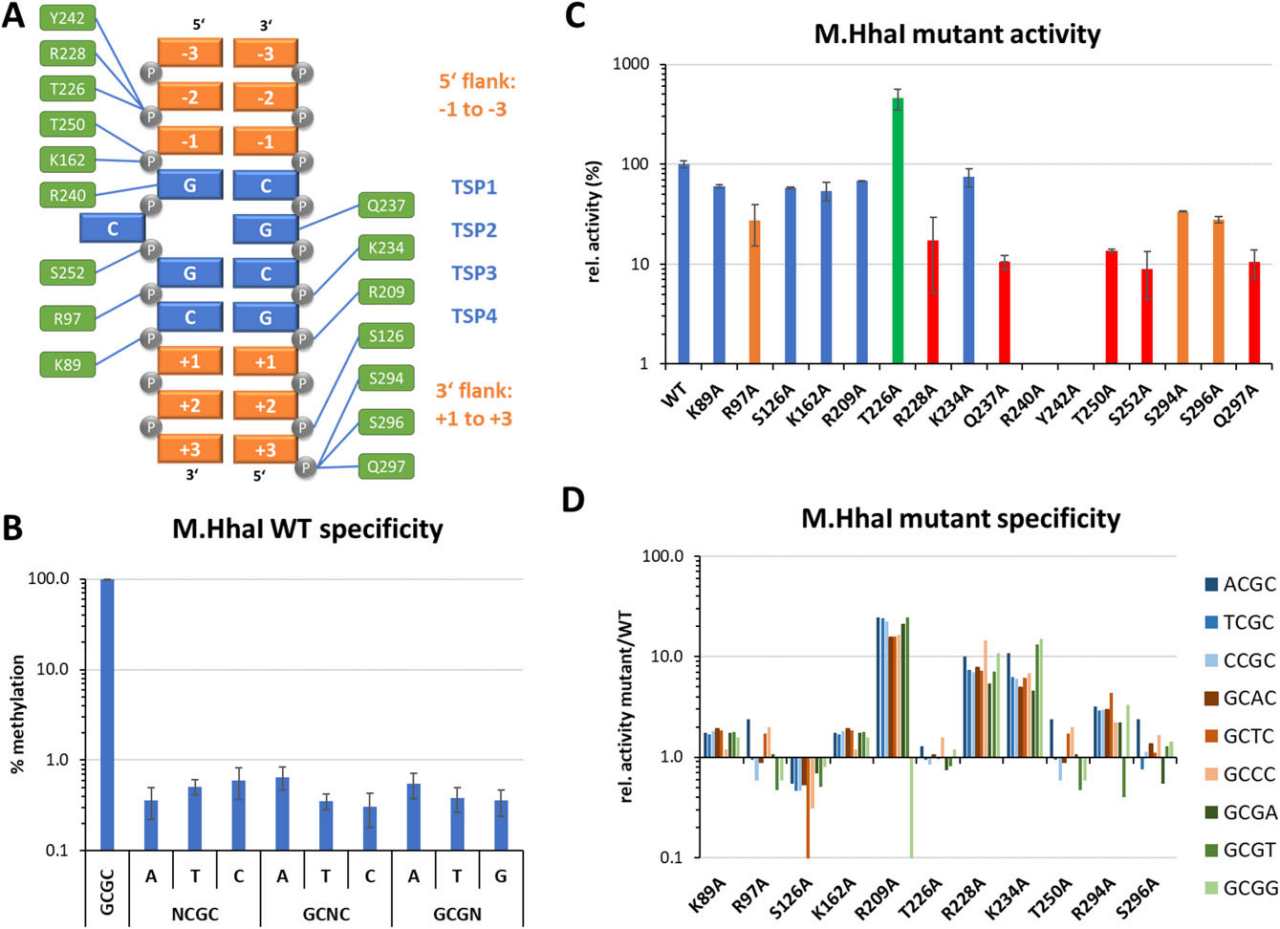
Specificity of M.HhaI WT and mutants. (**A**) Scheme of the sequence numbering and designations used in this work and the M.HhaI-DNA contacts analyzed by mutations. TSP, target site position. (**B**) Specificity of WT M.HhaI for GCGC methylation determined with the substrate library containing a CN site in randomized sequence context. Average methylation of GCGC and all near-cognate sites was determined. Error bars display the standard deviation (SD) of the independent experiments. Also see Supplementary Figs S6A and S7. (**C**) Activity of the M.HhaI mutants determined using a substrate library containing a GCGC site in a randomized sequence context by the radioactive DNA methylation assay. Error bars display SD of at least three experimental repeats. (**D**) Specificity of M.HhaI mutants for methylation of GCGC or near-cognate sites determined with the substrate library containing a CN site in randomized sequence context. Supplementary Fig. S7 shows the near-cognate site activity of the mutant relative to the GCGC activity divided by the corresponding value of the WT.

Our next aim was to investigate the DNA methylation specificity of M.HhaI, which modifies GCGC motifs (Fig. 3A). The enzyme was overexpressed from *E. coli* and purified (Supplementary Fig. S5). First, we aimed to investigate its specificity in GCGC DNA methylation. For this, the library of double stranded substrate DNA molecules containing a central CN site in a randomized sequence context was incubated with M.HhaI in 4 experimental repeats (Supplementary Table S1). Afterwards, the samples were bisulfite converted and sequenced. From the sequencing data, we extracted the reads containing GCGC sites, or one of its near-cognate variants, and determined their respective average methylation levels. In each experiment, high methylation was observed at the GCGC sequence and the relative methylation levels at near-cognate sites were highly correlated (Supplementary Fig. S6A). Hence the data were merged, showing a high specificity of M.HhaI for methylation at GCGC sites with methylation levels of all near-cognate sites well below 1% (highest value at GCAC with 0.6%) under conditions where the canonical site showed 98% methylation (Fig. 3B). Based on exponential rate fitting, these values correspond to a >2600-fold specificity of M.HhaI for the methylation of canonical GCGC sites. The high specificity of M.HhaI is also supported by the finding that under our experimental conditions no methylation was detectable at sites differing in more than one bp from GCGC.

**Figure 4.**
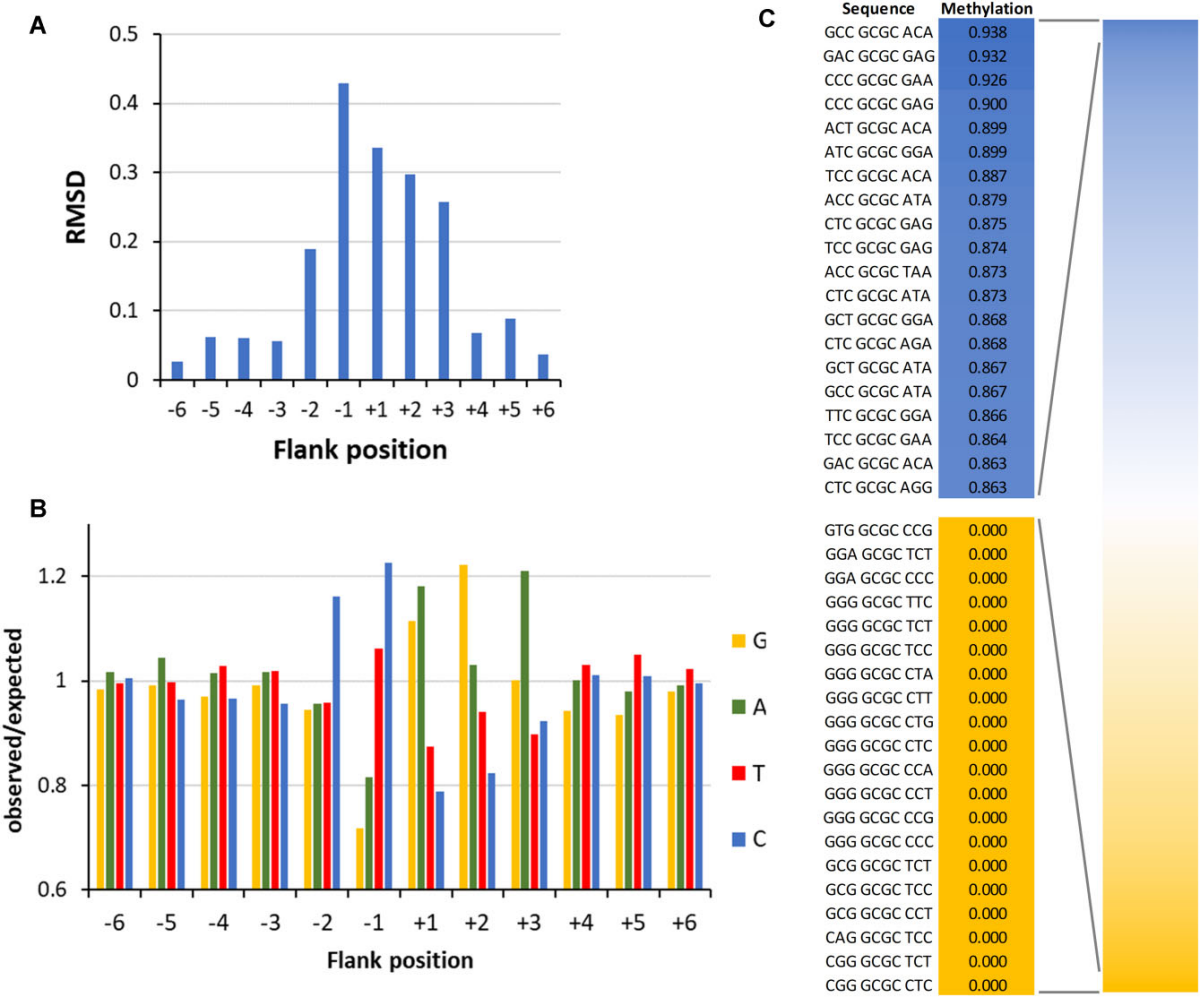
Flanking sequence preferences of M.HhaI determined with the substrate library containing a GCGC site in randomized sequence context. (**A**) Magnitude of the flanking sequence preferences for GCGC methylation at the −6 to +6 flank positions. Enrichment and depletion of bases at the positions in methylated substrate molecules were determined and the position-specific effect size plotted. Also see Supplementary Fig. S6B. (**B**) Enrichment and depletion of bases at the −6 to +6 flank positions in methylated substrate molecules were determined and plotted as o/e frequencies. (**C**) Average methylation levels of NNNGCGCNNN bins.

To investigate the flanking sequence preferences of M.HhaI, we prepared a substrate library containing GCGC sites in a randomized sequence context. It was incubated with M.HhaI in five experimental repeats, bisulfite converted and sequenced (Supplementary Table S1). From the sequencing data, we extracted the average methylation levels of NNGCGCNN sequences and found all of them highly correlated (Supplementary Fig. S6B). Hence, the replicates were combined and the o/e ratio of all nucleotides at the −6 to +6 flank sites were determined showing strongest flanking sequence effects at the −2 to +3 sites (Fig. 4A). Detailed analysis revealed that M.HhaI prefers C(−2), C(−1), A(+1), G(+1), G(+2), and A(+3) combined with particular disfavor for G(−1) (Fig. 4B). Hence, almost palindromic CC-GCGC-(GA)G core sequences are preferred. Quantitative analysis of average methylation levels in all 4096 NNNGCGCNNN sites (Fig. 4C) showed highest methylation of 93.8% in the GCC-GCGC-ACA context, which (as in M.SssI) is an example of a complex sequence effect, because a C(+2) is not among the preferred bases in the analysis focusing on individual flanking sites. In the same experiments, no methylation was observed in several NNNGCGCNNN bins all showing strong enrichment of disfavored bases. The sites with zero methylation had a combined sequencing coverage of 1149 reads, indicating that their methylation levels are below 0.26% (binomial distribution, p=0.05), which shows that M.HhaI has very strong flanking sequence preferences of >1000-fold. We also investigated flanking sequence preferences in the methylation of near-cognate sites by M.HhaI (Supplementary Fig. S7). Of note, due to the low methylation levels, these profiles are built on a relatively small number of methylated reads and should be interpreted only to show trends. Strikingly, in many cases the GCGC based flanking sequence preferences were drastically altered at near-cognate sites, which all showed site specific patterns. This observation suggests that the DNA conformations in the transition states of near-cognate sites methylation are distinct from the transition state DNA conformation of GCGC methylation, similarly as seen for non-CpG methylation of M.SssI.

### Specificity and flanking sequence preferences of the DNA methylation by M.HhaI mutants

To investigate the DNA interaction of M.HhaI in more mechanistic details, we inspected crystal structures of M.HhaI-DNA complexes [22, 23] and identified amino acid residues involved in direct base or phosphodiester backbone contacts (Table 1). The structures contain DNA molecules with a ATA-GCGC-TAT flanking sequence, which represents a weakly preferred M.HhaI preference (rank 1754 of 4096). The M.HhaI mutants were cloned, purified, and their concentrations were adjusted to the WT enzyme based on western-blot signals (Supplementary Fig. S5). First, the activity of the WT and mutant proteins were measured with the radioactive DNA methylation assay using the substrate with GCGC sites in randomized flanking sequence context (Fig. 3C). The data showed that R240A and Y242A were inactive. R240 forms a direct contact to the first bp of the target sequence (target site position 1, TSP1) that based on our data is essential for activity. Y242 likely has a role to stabilize R240 in its conformation. Five other mutants showed highly reduced activities (<20% relative activity when compared to WT M.HhaI): R228A, Q237A, T250A, and S252A, three of them (R228A, T250A, and S252A) affecting phosphodiester backbone contacts within the GCGC target site. Q237 inserts into the DNA at the place of the flipped cytosine. Q297A affects a backbone contact to the +3 flank. Three mutants showed a moderate reduction in activity with 20%–50% of relative activity: R97A, S294A, and S296A. R97 forms a backbone contact between GCGC TSP2/3, S294, and S296 form backbone contacts to the +2/+3 flank. Five mutants showed similar activities as the WT enzyme (>50% relative activity): K89A, S126A, K162A, R209A, and K234A which are all involved in backbone contacts at variable places. Finally, the activity of T226A was about four-times higher than that of WT M.HhaI suggesting that the backbone contact of T226 to the −1/−2 flank observed in the complex structures is not beneficial for catalysis.

**Table 1.** Compilation of M.HhaI mutants analyzed in this work and their biochemical effects

Mutation	Structural role	Activity	Specificity changes	Flanking sequence preference changes
K89A	Contact TSP4/flank +1	WT-like	-	Altered −2 preferences
R97A	Contact TSP3/4	Reduced	-	Altered preference at −2, −1, and +1
S126A	Contact −2/−3 flank	WT-like	Reduced methylation of GCTC	-
K162A	Contact TSP1/−1 flank	WT-like	-	-
R209A	Contact TSP4/+1 flank	WT-like	Reduced specificity, reduced methylation of GCGG	-
T226A	Contact −1/−2 flank	Increased	-	Altered −2 preferences
R228A	Contact −1/−2 flank	Strongly reduced	Reduced specificity	Altered −2 preferences
K234A	Contact TSP2/3	WT-like	Reduced specificity	-
Q237A	Inserts into the DNA at TSP2	Strongly reduced	n.d.	n.d.
R240A	Base contact TSP1	Inactive	n.d.	n.d.
Y242A	Contact −1/−2 flank	Inactive	n.d.	n.d.
T250A	Contact −1 flank/TSP1	Strongly reduced	-	Altered −1 and +1 preference
S252A	Contact TSP2/3	Strongly reduced	-	Altered −2 and +1 preferences
S294A	Contact +3/+4 flank	Reduced	-	-
S296A	Contact +2/+3 flank	Reduced	-	-
Q297A	Contact +3/+4 flank	Strongly reduced	n.d.	n.d.

TSP, target site position; -, no strong changes; n.d., not determined (because of insufficient activity).

The activity of 11 of the mutants was sufficient to investigate their specificities for methylation in the GCGC context revealing high preferences and small to moderate deviations from the WT pattern in most cases (Fig. 3D). However, in four cases strong (>10-fold) specificity changes were observed. Two mutations, R228A and K234A, caused a global reduction of specificity indicating that their DNA backbone contacts to the −1/−2 flank and TSP 3/4 are important to establish an efficient transition state conformation with the GCGC sequence, but not for the methylation of near-cognate sites. An even stronger global reduction of specificity was detected with R209A, which contacts the phosphodiester group between TSP4 and the +1 flank. Interestingly, in this case a strongly reduced methylation of GCGG (a near-cognate site with C to G exchange at TSP4) was observed which is going against the global trend of this mutant to show reduced specificity. This specific effect matches with the DNA-contact of R209 at TSP4 indicating that R209 is required for methylation of this near-cognate site. If GCGG methylation might involve alternative enzyme–DNA contact patterns than observed with the canonical GCGC sequence awaits further structural studies. In particular it will be interesting to investigate, if a direct contact of R209 to the G at TSP4 is formed during methlytion of GCGG near-cognate sites. Finally, S126A led to a very defined loss of GCTC methylation indicating that S126 is essential for the methylation of this near-cognate site. This result does not directly correlate with the structural data, because S126 forms a contact to the phosphodiester group at the −2/−3 flank.

**Figure 5.**
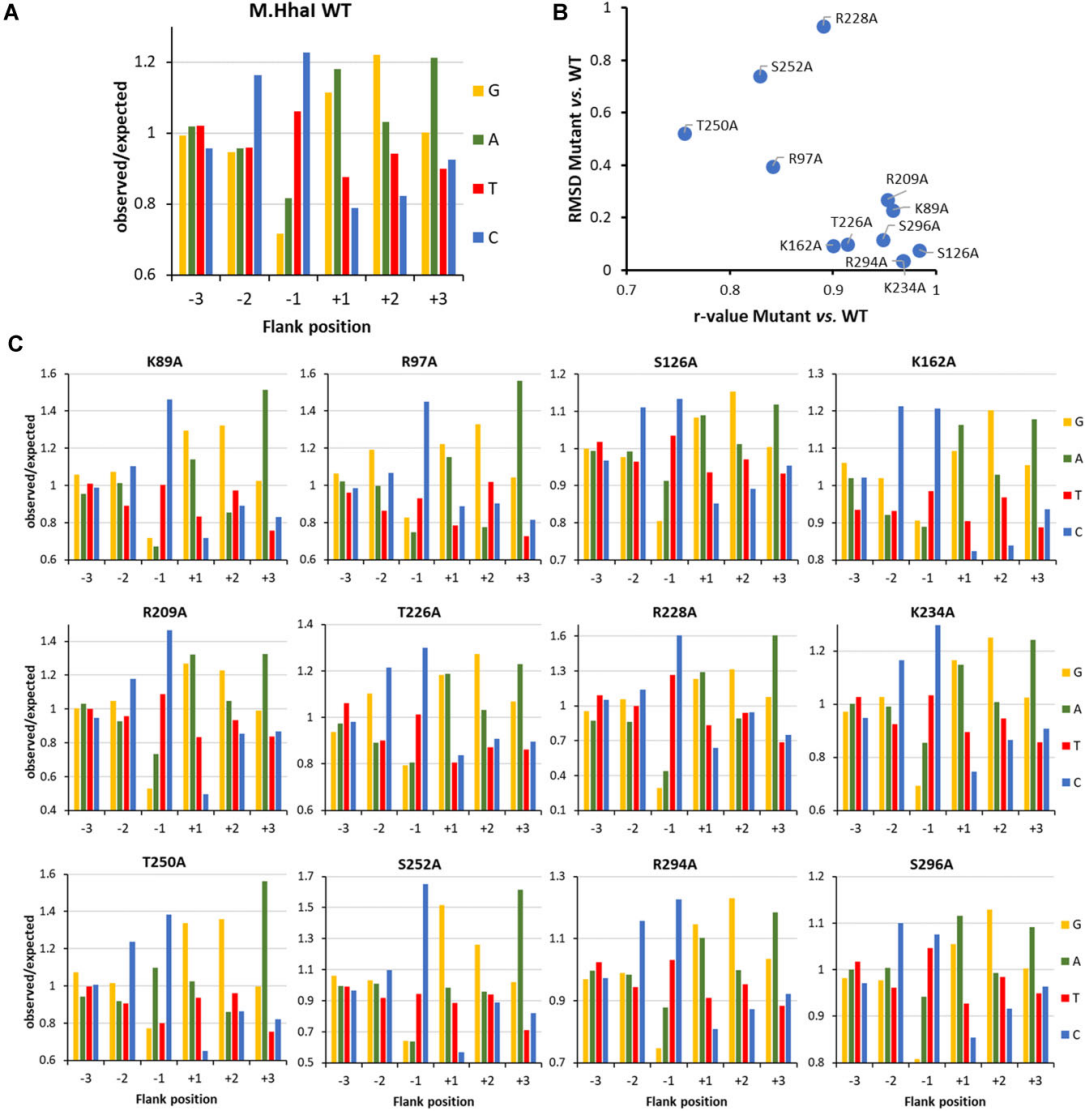
Flanking sequence preferences of M.HhaI mutants determined with the substrate library containing a GCGC site in randomized sequence context. (**A**) Sequence preferences of WT M.HhaI at the −3 to +3 flank positions plotted as o/e frequencies. Taken from Fig. 4B for comparison. (**B**) Quantitative differences of the −6 to +6 flanking sequence preferences of M.HhaI mutants versus WT expressed as Pearson correlation (*r*-value) and RMSD. (**C**) Flanking sequence preferences of M.HhaI mutants at the −3 to +3 flank positions plotted as o/e frequencies.

Finally, the flanking sequence preferences for GCGC methylation were analyzed for 12 of the M.HhaI mutants with sufficient activities (Fig. 5). For comparison of the mutants’ effects, the correlation (Pearson *r*-value) of the WT and mutant preference profiles as well as the absolute difference in the flanking sequence preference profiles measured in form of root mean square deviation (RMSD) values were determined. This analysis identified four mutants with strongest effects T250A, S22S, R228A, and R97A, visible as low *r*-values and high RMSDs in the comparison of WT and mutant preferences. Of note, three of them were among the mutants with reduced activity and R228A also showed relaxed GCGC specificity. Detailed comparison of the WT and mutant preference profiles revealed several interesting position and mutant specific effects:

−2 flank, WT preference C > (GAT): At this site, many mutants showed a similar change of the preferences towards a (CG) > A > T pattern (K89A, R97A, R209A, T226A, R228A, T250A, S252A).
−1 flank, WT preference C > T > A > G: At this site, T250A showed a strong elevation of A(−1) preference yielding a C > A > (GT) profile. This finding is in agreement with the contact of T250 to the TSP1/−1 flank phosphodiester group.
- +1 flank, WT preference (GA) > T > C: Several of the mutants displayed an increase of the G(+1) preference yielding a G > A > (TC) preference profile. This change occurred to a variable degree, most strongly with T250A and S252A, though both residues do not directly contact this DNA region.
- +2 flank, WT preference G > A > (TC): At this site, only moderate changes of the WT preference profile were observed with R97A and T250A, which both showed a drop of A(+2) preference yielding a G > (TC) > A profile. In case of R97A, this agrees with the contact of R97 to the phosphodiester group between TSP3/4.
- +3 flank, WT preference A > (GCT): Strikingly, the WT preference was not altered in any of the mutants although several of them affect DNA backbone contacts to the +3 flank site (S126A, S294A, S296A). This result suggests that A(+3) plays an important role in the M.HhaI-DNA interaction and interference with this contact could explain the strong reduction in activity of the Q297A mutant.

In summary, numerous alterations of position specific flanking sequence preferences were observed with the M.HhaI mutants. In some cases, these changes could be correlated to phosphodiester backbone contacts of the mutated residues next to the site of altered flanking sequence preferences suggesting that the corresponding contact could be involved in the readout of flanking sequence specific DNA conformations. In other cases, the results could not be rationalized based on the available structural data. The uniformity of the altered flanking sequence preferences profiles at the −2 and +1 sites observed with many mutants is surprising because different mutations showed similar effects although they contact different parts of the DNA sequence. This observation could be explained if alternative transition state conformations of the enzyme–DNA complex exist and alteration of enzyme–DNA contacts can differentially affect the occupancy of these alternative conformational states.

### Specificity and flanking sequence preferences of the DNA methylation by M.HaeIII

**Figure 6.**
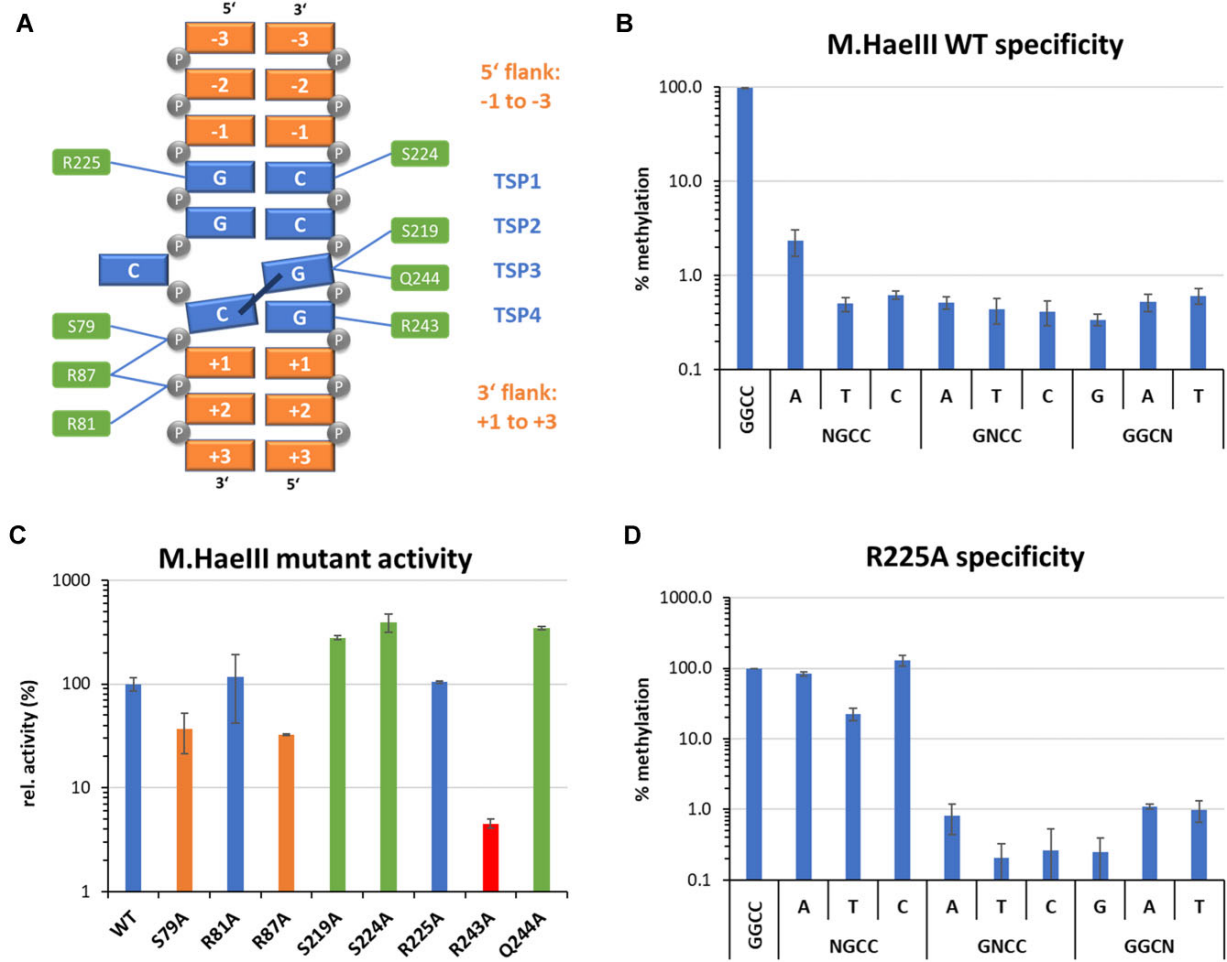
Specificity of M.HaeIII WT and mutants. (**A**) Scheme of the sequence numbering and designations used in this work and the M.HaeIII–DNA contacts analyzed by mutations. TSP, target site position. (**B**) Specificity of WT M.HaeIII in GGCC methylation determined with the substrate library containing a CN site in randomized sequence context. Average methylation of GGCC and all near-cognate sites was determined. Error bars display the SD of the independent experiments. Also see Supplementary Fig. S8A. (**C**) Activity of the M.HaeIII mutants determined using the substrate library containing a GGCC site in randomized sequence context by the radioactive DNA methylation assay. Error bars display SD of at least three experimental repeats. (**D**) Specificity of the M.HaeIII R225A mutant shown as described in panel (B). Also see Supplementary Fig. S8C for the specificities of the other M.HaeIII mutants.

Next, we studied the specificity of DNA methylation by M.HaeIII which modifies GGCC motifs (Fig. 6A). The enzyme was overexpressed in *E. coli* and purified (Supplementary Fig. S3B). For investigation of its specificity in GGCC DNA methylation, the library of double stranded substrate DNA molecules containing one central CN target site in a randomized flanking sequence context was incubated with M.HaeIII in six experimental repeats (Supplementary Table S1). Afterward, the samples were bisulfite converted and sequenced as described above. From the sequencing data, we extracted the reads containing the GGCC site, or one of its near-cognate variants, and determined their respective average methylation levels. In each experiment, high methylation was observed at the target sequence and the relative methylation levels at near-cognate sites were highly correlated (Supplementary Fig. S8A). Hence the data were merged, showing a high specificity of M.HaeIII with methylation levels of most near-cognate sites well below 1%, with the exception of AGCC which showed 2.3% of methylation (Fig. 6B). Based on exponential rate fitting, these values correspond to a >670-fold specificity of M.HaeIII for the methylation of GGCC over AGCC sites and preferences >2500-fold for all other near-cognate sites. Similarly as in the case of M.HhaI, no methylation was detectable at sites differing in more than one bp from GGCC.

**Figure 7.**
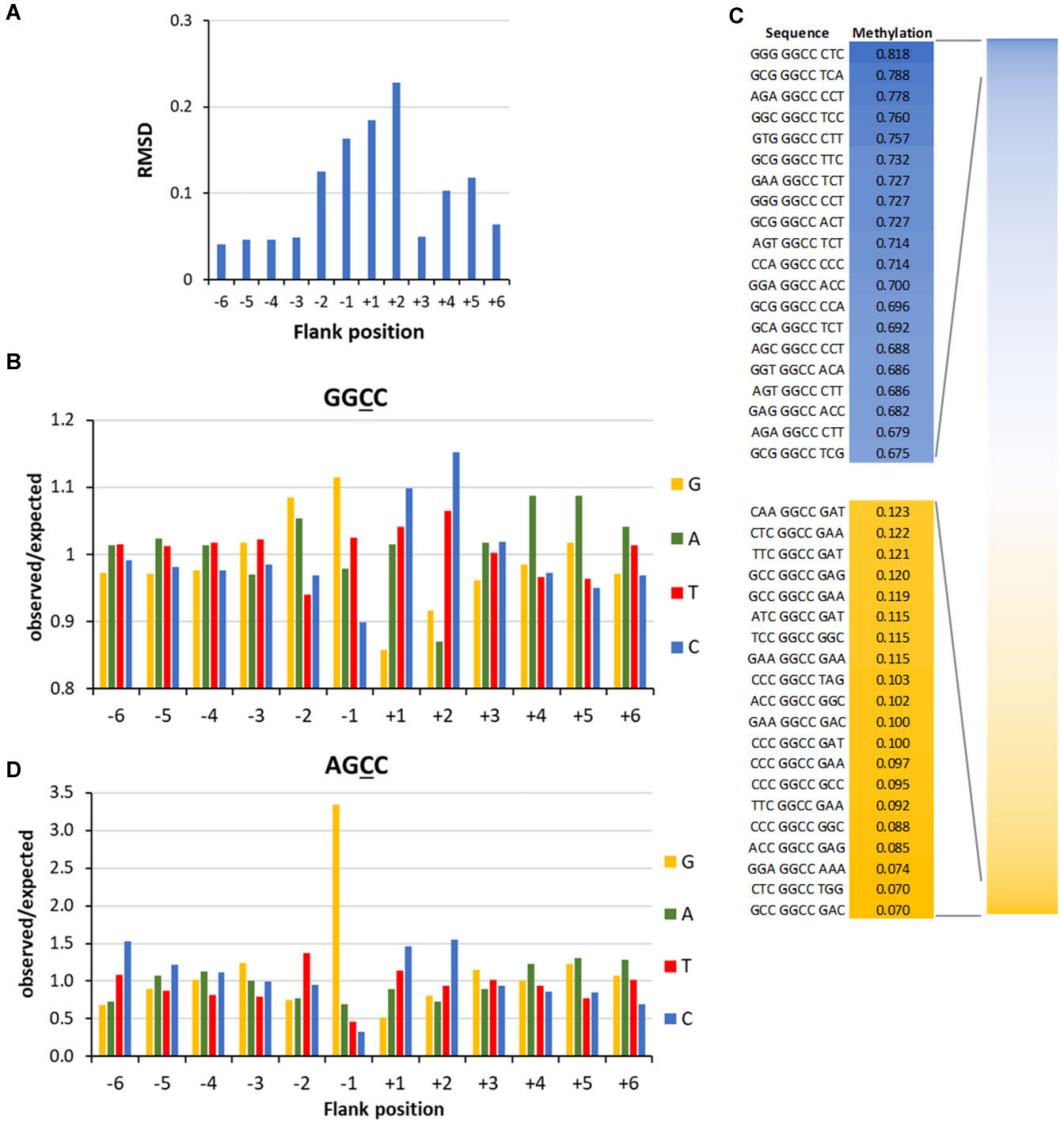
Flanking sequence preferences of M.HaeIII. (**A**) Magnitude of the flanking sequence preferences for GGCC methylation at the −6 to +6 flank positions. Enrichment and depletion of bases at the positions in methylated substrate molecules were determined and the position-specific effect size plotted. Also see Supplementary Fig. S8B. (**B**) Enrichment and depletion of bases at the −6 to +6 flank positions in methylated substrate molecules were determined and plotted as o/e frequencies. (**C**) Average methylation levels of NNNGGCCNNN bins. (**D**) Flanking sequence preferences of M.HaeIII for methylation in AGCC context, taken from the methylation data obtained with the substrate library containing a CN site in randomized sequence context.

To investigate the flanking sequence preferences of M.HaeIII, methylation reactions were conducted using a GGCC substrate in a randomized flanking sequence context in five independent experimental repeats (Supplementary Table S1). Average methylation levels were determined in all 256 NNGGCCNN bins and they were found to be highly correlated (Supplementary Fig. S8B). Hence, the data were combined. The −6 to +6 flanking sequence preference analysis revealed strong preferences at the −2 to +2 as well as +4 and +5 sites (Fig. 7A) including preferences for G(−2) and A(−2), G(−1), C(+1), C(+2), T(+2), A(+4), and A(+5) combined with disfavor for C(−1), G(+1), G(+2), and A(+2) (Fig. 7B). Interestingly, the palindromic GG-GGCC-CC sequence context was most preferred while the complementary sequence CC-GGCC-GG was most disfavored.

The preference for A at the +4 and +5 flanking sites is interesting, because these sites are not contacted in the crystal structure. A similar finding has been made with DNMT3A which prefers A and T at the +5 to +8 position relative to the CpG target site [57]. This enzyme interacts as a dimer of catalytically active subunits with two CpG sites on one DNA molecule and thereby introduces DNA bending of approximately 40° [58]. The AT preference was observed at the region of maximal bending in agreement with the fact that AT-rich DNA shows a higher flexibility. While it is not known if M.HaeIII dimerizes on DNA it has been demonstrated that M.HaeIII dimers are active [59], suggesting that a similar DNA bending model may explain its preferences for A at the +4 and +5 flanking sites.

Analysis of average methylation levels of the 4096 NNNGGCCNNN sites (Fig. 7C) revealed a 17-fold ratio in the initial methylation rates of the best and worst flanking sites. However, it needs to be noticed that the effects of the +4 and +5 flank sites could not be included in this analysis, suggesting that the overall flanking sequence effects of M.HaeIII are in a similar range as those observed for M.SssI and M.HhaI. We also inspected the flanking sequences of near-cognate site methylation. However, due to the low methylation levels and lower sequencing coverage, in most cases only few methylation events were observed, not permitting a reliable analysis. Only in the case of AGCC, the methylation was strong enough to allow a flanking sequence analysis which revealed that flanking sequence preferences were completely different from those observed in the GGCC context. Methylation of AGCC sites was only possible in a G(−1) sequence context while G(−1) is only moderately preferred in the context of GGCC methylation (Fig. 7D). Additionally, the −2 preferences of AGCC and GGCC methylation were fundamentally different, while patterns at the plus-side were comparable. This observation agrees with the findings made with M.SssI and M.HhaI. It supports our model that the transition state DNA conformations for methylation of the cognate and near-cognate MTase sites are different. In case of H.HaeIII, it is likely that these strong changes of minus-side preferences reflect a target site and flanking sequence specific conformational change of the DNA after base flipping in the AGCC context.

### Activity of M.HaeIII mutants

To investigate the DNA interaction of M.HaeIII in more mechanistic details, we inspected the crystal structure of the M.HaeIII–DNA complex [27] and identified amino acid residues involved in direct base or phosphodiester backbone contacts (Table 2). The structure was generated with a DNA molecule with a GCA-GGCC-ACC flanking sequence, which represents a moderate good M.HaeIII preference (rank 728 of 4096). The mutants were cloned, purified, and their concentrations were determined (Supplementary Fig. S3B). The catalytic activities of the mutant were determined with the radioactive DNA methylation assay using the substrate with a single GGCC site in a randomized flanking sequence context (Fig. 6C). The data showed that R243A has a strongly reduced activity (<20% relative activity when compared to WT M.HaeIII) indicating that the contacts of this residue to the TSP4 and +1 flank are important for catalysis. The S79A and R87A mutants showed moderately reduced activities (20%–50% of relative activity). Both are involved in contacts to phosphodiester groups between TSP4 and the +1 flank and the +1/+2 flank indicating that the anchorage of TSP4 (which is involved in the structural rearrangement of the DNA after base flipping) and the adjacent flanking DNA is important for DNA methylation by M.HaeIII. However, two mutants showed a WT-like activity with >50% relative activity (R81A and R225A) and three even showed an increase in activity (S219A, S224A, and Q244A). These findings cannot be rationalized in the context of the M.HaeIII–DNA structure, because S219, S224, R225, and Q244 all are engaged in direct base contacts at the target site in the enzyme–DNA complex and they will be discussed later.

**Table 2.** Compilation of M.HaeIII mutants analyzed in this work and their biochemical effects

Mutation	Structural role	Activity	Specificity changes	Flanking sequence preference changes
S79A	Contact TSP4/+1 flank	Reduced	-	-
R81A	Contact +1/+2 flank	WT-like	-	Altered +1 preferences
R87A	Contacts +1/+2 flank, TSP4/+1 flank	Reduced	-	Altered +1 preferences
S219A	Contact TSP3	Increased	-	Altered +1 preferences
S224A	Contact TSP2	Increased	-	Altered −1 and +1 preferences
R225A	Contact TSP1	WT-like	Loss of TSP1 recognition	Altered −2 and −1 preferences
R243A	Contact TSP4, Flank +1	Strongly reduced	-	Altered −1 and +1 preferences
Q244A	Contact TSP3	Increased	-	Altered +1 preference

TSP, target site position; -, no strong changes.

### Specificity and flanking sequence preferences of the DNA methylation by M.HaeIII mutants

The specificities of all M.HaeIII mutants for methylation in the GGCC context were analyzed revealing small to moderate effects in most cases (Supplementary Fig. S8C), again illustrating that individual base contacts surprisingly are not critical for DNA recognition by M.HaeIII. Only R225A showed a strong loss of sequence recognition at the first bp of the target site (Fig. 6D) which is in line with the contact of this residue to TSP1 and indicates that this contact is required for the readout of a G at TSP1. However, based on the WT-like catalytic activity of R225A, this contact is not supportive for catalysis, suggesting that the role of R225 is to reduce enzyme activity if non-GC base pairs are situated TSP1, an interesting mechanism of negative specificity whose details await further structural and biochemical studies. Evidence for a high relevance of R225 in the recognition of TSP1 had been provided already previously in an experiment based on random mutagenesis followed by *in vitro* selection for M.HaeIII mutants with elevated AGCC methylation activity [31].

**Figure 8.**
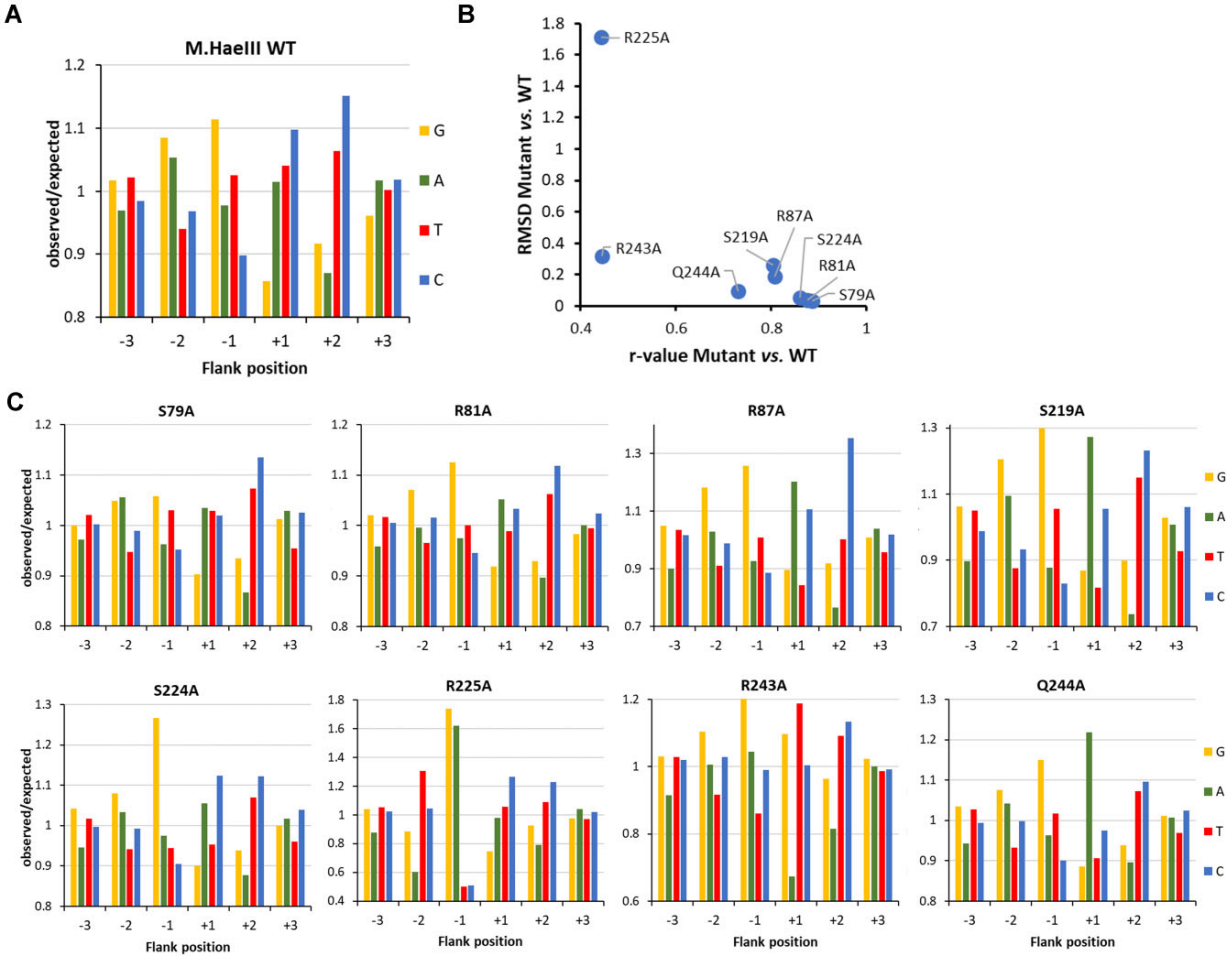
Flanking sequence preferences of M.HaeIII mutants determined using the substrate library containing a GGCC site in randomized sequence context. (**A**) Sequence preferences of WT M.HaeIII at the −3 to +3 flank positions plotted as o/e frequencies. Taken from Fig. 7B for comparison. (**B**) Quantitative differences of the −6 to +6 flanking sequence preferences of M.HaeIII mutants versus WT expressed as Pearson correlation (*r*-value) and RMSD. (**C**) Flanking sequence preferences of M.HaeIII mutants at the −3 to +3 flank positions plotted as o/e frequencies.

Next, the flanking sequence preferences of the M.HaeIII mutants for GGCC methylation were analyzed and compared with the WT preferences on the basis of the Pearson *r*-values and RMSDs (Fig. 8). This analysis identified two mutants with strong effects (R243A and R225A), and three more with moderate effects (Q244A, S219A, R87A) (Fig. 8B). Similarly as with M.HhaI, flanking sequence preference analysis of the mutants revealed several interesting position and mutant specific effects:

−2 site, WT preference G > A > (CT): R225A showed a strong effect with a new preference profile of T > C > G > A.
−1 site, WT preference G > T > A > C: S224A and R243A showed a reduction in T(−1) preference and R225A showed elevated preference for G(−1) or A(−1).
- +1 site, WT preference C > (TA) > G: R243A (T > GC) >> A and Q244A (A >> CTG) showed completely new preferences. Three mutants showed an elevation of A(+1) preference to a variable degree: R87A, S219A, and S224A.
- +2 site, WT preference C > T > G > A: No strong changes in flanking sequence preferences were observed at this site.

In some cases, correlations could be drawn between the contacts disrupted by the mutations and the changes in flanking sequence preferences, as for example in the case of R243A which contacts TSP4 and the +1 flank and has a completely altered +1 site preference pattern. In other cases, strong effects were not correlated with DNA contacts as in the case of R225A (change of the −2 site preference, but contact to the TSP1), or Q244A (change in +1 site preference, but contact to TSP3). The observation that three distinct preference profiles were observed with groups of mutants at the +1 site (one WT-like, one R243A like and one with elevated A preference) indicates that different enzyme–DNA conformations can lead to productive transition states for the methylation of GGCC sites supporting a similar conclusion based on the M.HhaI mutant data.

### Transition state structure of the DNA MTase reaction

Two of the M.HaeIII mutants showed a WT-like activity and three more mutants even showed an elevated activity when compared with M.HaeIII WT. This was unexpected because all of the mutated residues were involved in contacts in the M.HaeIII–DNA complex that appear highly relevant. Moreover, with M.HhaI and M.HaeIII we observed several examples of mutants with clear changes in the specificity profiles and flanking sequence preferences that could not be rationalized based on of the crystal structures. To explain these striking observations, we suggest that the transition state of MTase catalysis differs from the structurally analyzed covalent DNA–enzyme adduct with flipped target base. To introduce this model, it is relevant to consider that the DNA methylation reaction includes three distinct chemical steps [3]:

- Step 1: The first chemical reaction is the nucleophilic attack of the active site Cys residue on the C6 position of the flipped target base. Critical steps preceding this reaction are the binding of the enzyme to the target sequence in double stranded DNA, target base flipping, and placement of the flipped base in the active site pocket of the enzyme.
- Step 2: Step 1 is followed by the methyl group transfer from AdoMet to the cytosine C5 atom leading to the conformation seen in many structurally resolved complexes of MTases.
- Step 3: Finally, elimination of the Cys combined with deprotonation at C5 occurs, allowing the methylated base to rotate back into the DNA.

Based on chemical considerations, we argue that the kinetically limiting step of the MTase reaction is step 1. This assumption is plausible, because the catalytic Cys residue is expected to be protonated most of the time, the flipped state of the base has a very low occupancy, and very accurate positioning of the flipped base with respect to the Cys is required, all suggesting that this step is slow. In contrast, the methyl group is transferred from a donor with very high group transfer potential (AdoMet) to a highly activated nucleobase in step 2, implying that this reaction should be very fast. Similarly, step 3 which re-establishes aromaticity is expected to proceed fast, and (anyway) this step is invisible in our methylation assay as methyl group transfer has already taken place. As mentioned above, step 1 of the methylation reaction included several sub-steps and it is unclear which of them is rate-limiting with each enzyme, each enzyme mutant, and each substrate. This implies that the transition state of this reaction is somewhere in between double stranded DNA before base flipping and the structure of the structurally resolved covalent complex. Hence, it is well conceivable that other enzyme–DNA contacts are formed in the transition state conformation than the ones established in the covalent complex with a flipped base, depending on the enzyme, enzyme mutant, and perhaps even the substrate sequence.

### Specificity and flanking sequence preferences of additional bacterial DNA-(cytosine C5)-MTases

**Figure 9.**
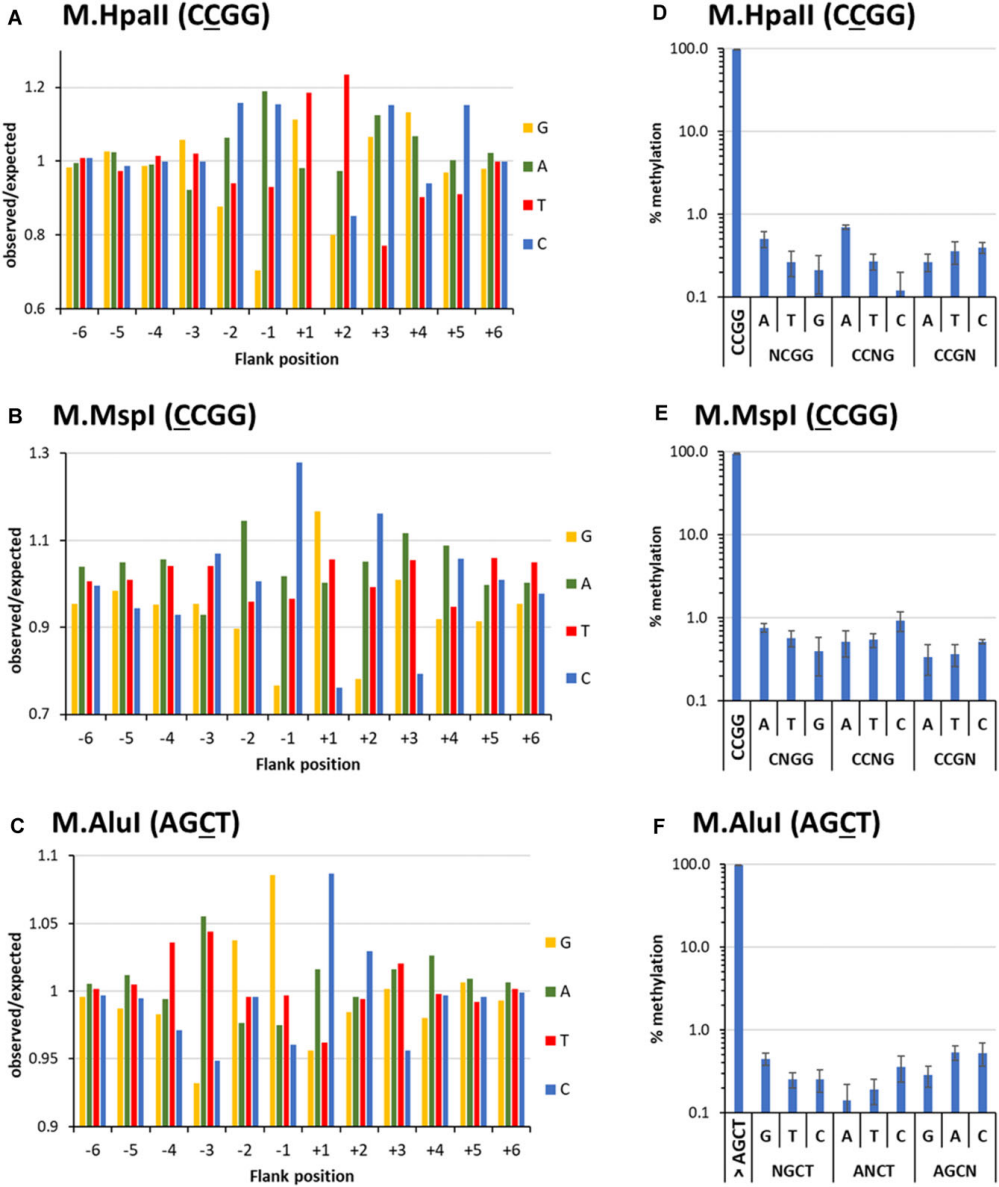
Flanking sequence preference and specificity data of M.HpaII, M.MspI, and M.AluI. (**A–C**) Flanking sequence preferences of M.HpaII, M.MspI, and M.AluI determined by methylation of pools of DNA molecules containing a CCGG or AGCT site in randomized sequence context. Enrichment and depletion of bases at the −6 to +6 flank positions in methylated substrate molecules were determined and plotted as o/e frequencies. Also see Supplementary Figs S9 and S10. (**D–F**) Specificities of M.HpaII, M.MspI, and M.AluI for methylation of their target motifs determined with the pool of DNA molecules containing a CN site in randomized sequence context. Average methylation of the corresponding target sites and all near-cognate sites were determined. Error bars display the SD of the independent experiments.

Finally, the flanking sequence preferences and specificities were analyzed for three additional bacterial DNA-(cytosine C5)-MTases, *viz*. M.HpaII (CCGG) (Supplementary Fig. S3C) and the commercially obtained M.MspI (CCGG) and M.AluI (AGCT) enzymes. M.HpaII and M.MspI have a common CCGG target site but methylate different cytosine residues within it. M.AluI was studied as an example of an enzyme with a more AT-rich target sequence. In each case several methylation reactions were conducted (Supplementary Table S1). Data of corresponding experiments were highly correlated and the data sets were merged (Supplementary Fig. S9). Analysis of the flanking sequence preferences (Fig. 9A) revealed that M.HpaII (CCGG) prefers (AC) (AC)-CCGG-(GT)T (CAG) sequences. Hence, it favors amino groups in the major groove of the DNA at the +1 and +2 flank position. M.MspI (CCGG) prefers AC-CCGG-(GT) (CA) (ATG) (Fig. 9B), which resembles the preferences of M.HpaI suggesting a similar mode of DNA interaction. Finally, M. AluI (AGCT) shows a T (AT)GG-AGCT-CC preference with strong flanking sequence effects up to the −4 position (Fig. 9C). Average methylation levels of NNNXNNN bins (Supplementary Fig. S10) in each case revealed flanking sequence effects of 85-fold or more considering the sequencing depths. All three enzymes showed high specificities for methylation in their target sequence with methylation levels of near-cognate sites <1% in all cases (Fig. 9D–F).

### Correlation of flanking sequence preferences and DNA shape parameters

**Figure 10.**
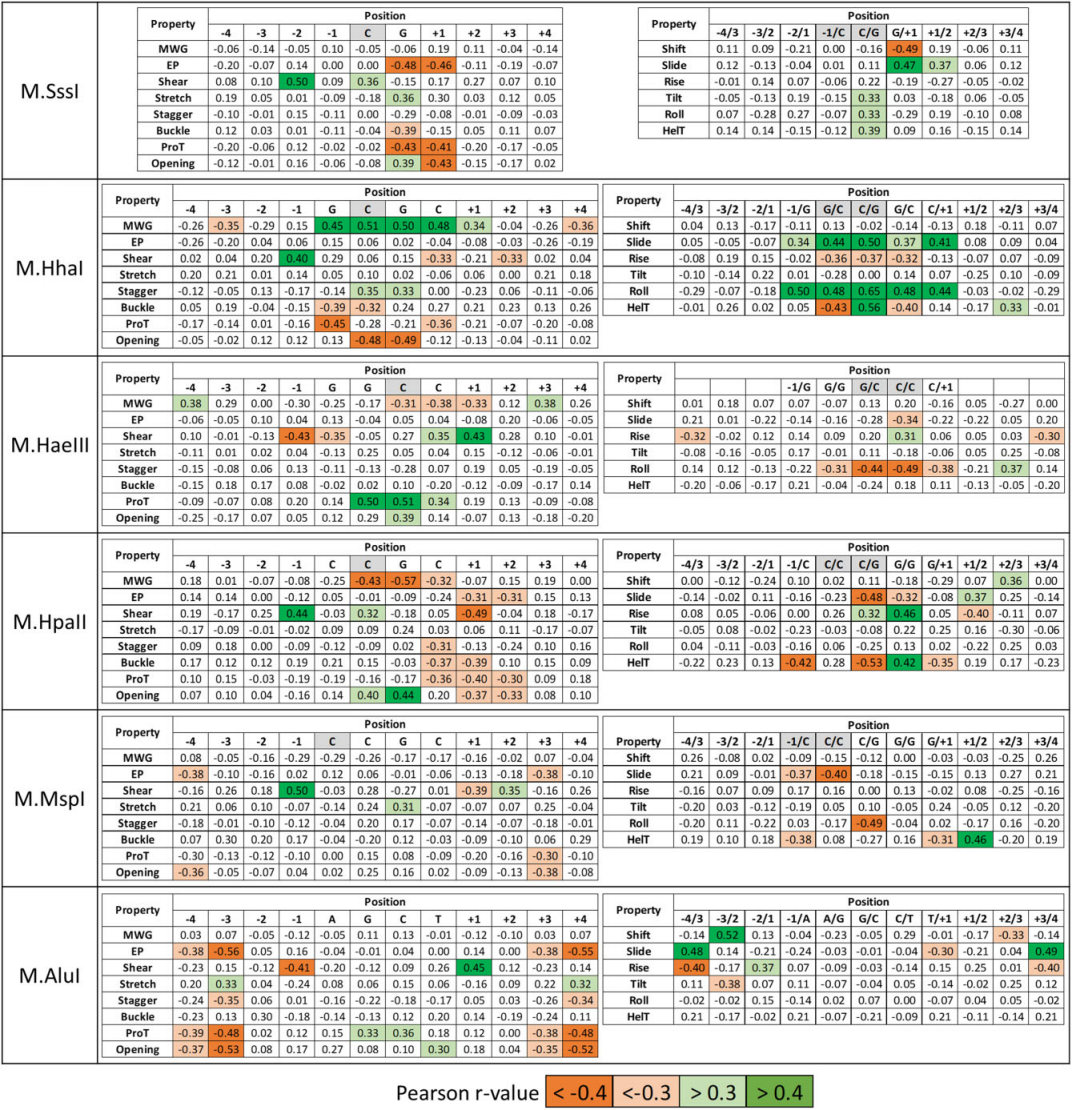
Correlation of flanking sequence preferences and flanking sequences dependence of DNA shape parameters. Enzyme activities were calculated for all N_4_XN_4_ sequences (where X denotes the specific recognition sequence of each enzyme). DNA shape parameters were predicted for the same sequences using the Deep DNAshape server (https://deepdnashape.usc.edu/) [51, 52] and both data sets were correlated. The figure shows the pairwise Pearson *r*-values, the color code is provided at the bottom. The shape properties are explained in Supplementary Fig. S1.

Proteins recognize DNA sequences by direct hydrogen-bonding contacts mainly in the major groove of the DNA (direct readout). However, they also form a tight interaction network with the DNA backbone thereby enforcing a specific DNA conformation in the protein–DNA complex [8, 9] and in case of enzymes, in the transition state of the catalyzed reaction. Of note, the intrinsic DNA sequence dependent DNA shape can favor or disfavor the DNA conformation required in the protein–DNA complex. The intrinsic DNA conformation changes with the DNA sequence in a complex manner and at sites up to 4 bp away from a particular position. Therefore, we next explored if the flanking sequences preferences of DNA MTases show noticeable correlations with sequence dependent DNA shape features (Supplementary Fig. S1). For this, DNA shape features were predicted for all N4XN4 sequences for all enzymes (where X denotes the MTase target sequence) using the Deep DNAshape server (https://deepdnashape.usc.edu/) [51, 52]. Relative methylation activities of all enzymes were calculated for all N_4_XN_4_ sequences on the basis of the position specific enrichment or depletion of nucleotides in the flanking sequence preference profiles. Both steps are described in the Materials and Methods section in detail. The activity and DNA shape data for the N_4_XN_4_ sequences were then correlated and Pearson *r*-values were determined for the 14 different DNA shape properties (Fig. 10). Strikingly, with each enzyme at least three shape properties showed strong correlations or anticorrelations with the flanking sequence preferences with *r*-values above 0.4 or below −0.4 and many more showed correlations above 0.3 or below -0.3. This suggests that in these cases flanking sequence preferences affect enzyme activities through their influence on the corresponding DNA shape property. For example, in case of M.HhaI MGW of TSP1-4 is strongly correlated with flanking sequence preferences while Opening is anticorrelated at TSP2 and 3 (Fig. 10). This implies that flanking sequences that increase the MGW at TSP1-4 and/or decrease Opening at TSP 2 and 3 are favorable for activity.

As shown in Fig. 10, correlations and anticorrelations between sequence dependent variations of the DNA shape and flanking sequence preferences were regularly observed at the target sequence, but in many cases also in the flanks, particularly the ± 1 flanking site. However, strong correlations were also observed at farther flanks, most strikingly with M.AluI (−4, −3, +3, and +4) and also M.MspI (−4 and +3) suggesting that these enzymes tightly contact the outer parts of the bound DNA which is in line with their flanking sequence preferences showing strong effects at the ± 3 and ± 4 flank regions (Fig. 9). In summary, our analysis suggests that the sequence dependent modulation of DNA conformations is an important mechanism mediating DNA MTase flanking sequence preferences. Furthermore, it provides direct insights into the site and shape parameter specific preferences of transition state DNA conformations of the different enzymes.

**Figure 11.**
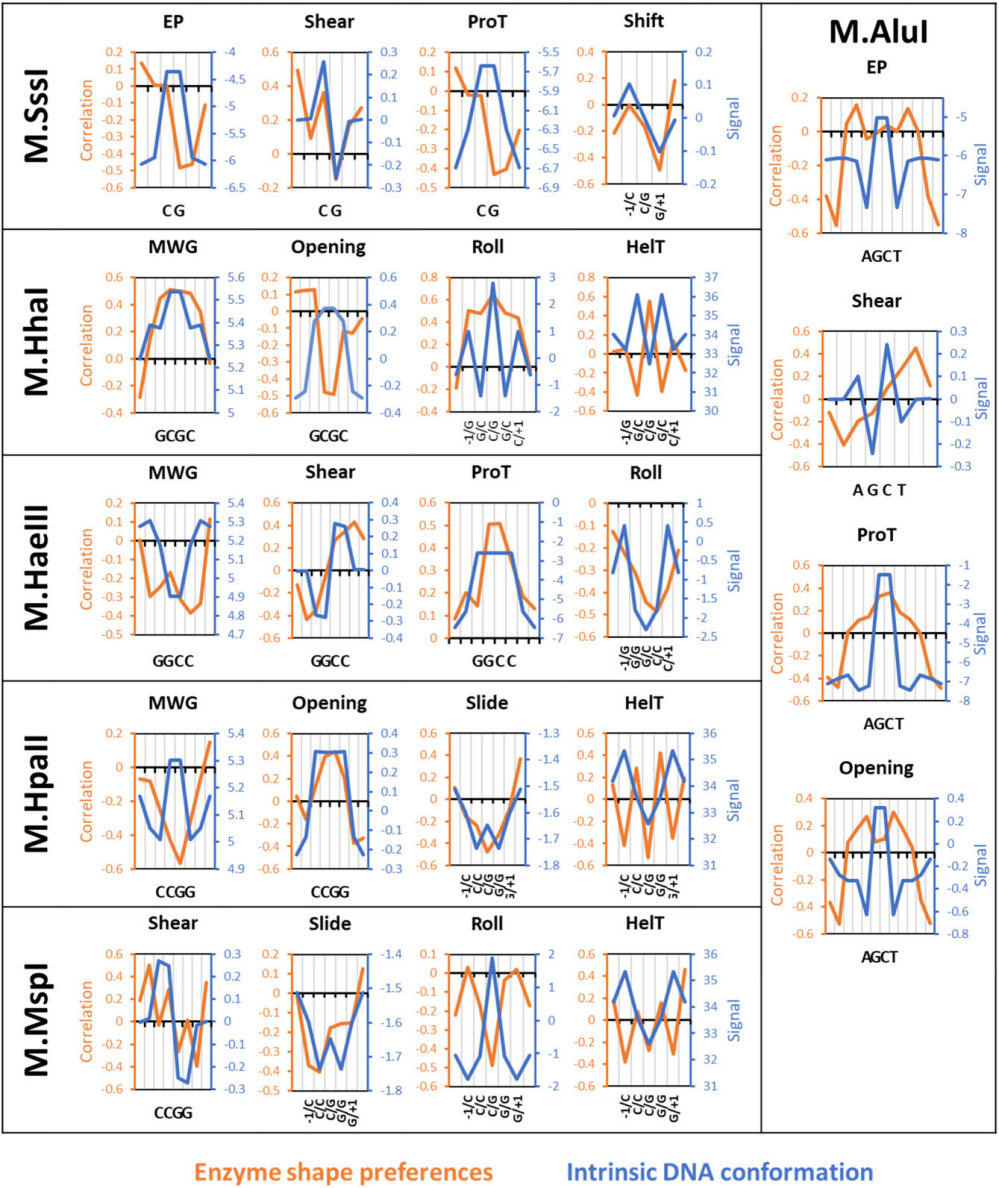
Examples of the correlation of individual shape preferences with the intrinsic DNA shape parameters of the enzyme’s target sequence. The individual shape preferences of enzymes are expressed as the correlation of individual shape properties with enzyme activity (*r*-value) on the left axis. The *r*-values of all correlations are provided in Fig. 10. The intrinsic DNA shape parameters of the target sequence are expressed in property dependent units on the right axis. All intrinsic shape analyses are provided in Supplementary Fig. S11.

In the next step, we determined the intrinsic DNA shape parameters of the different target sequences from the DNA shape profiles averaged over all flanking sequences (Supplementary Fig. S11) and compared the MTase shape preferences with the intrinsic target sequence shape (Fig. 11). In many cases, enzyme preferences and intrinsic DNA shape properties were positively correlated indicating that the intrinsically preferred DNA conformation is favorable for activity and flanking sequences supporting the adoption of this conformation lead to an elevated DNA methylation rate. For example, M.SssI shows a high Shear value at the C(TSP1) and a low value at G(TSP2). It shows a positive correlation of shape parameters and activity at the C, but a negative correlation at the G. Hence large Shear values at C and low shear values at G are favorable for catalysis, indicating that the intrinsic Shear preferences of the target CpG sequence support catalysis. In some examples, positive correlations of enzyme DNA shape preferences and intrinsic shape properties reach over the entire target sequence, as seen for Shift in M.SssI, MWG in M.HhaI, Slide in M.HpaII, or ProT in M.HaeII.

However, in other cases anticorrelations were observed between enzymatic shape preferences and intrinsic DNA shape properties. In these cases, the enzyme needs to overcome the intrinsically preferred DNA conformation of the target sequence to reach the transition state conformation. Hence target sites with flanking sequences acting against the intrinsic shape preferences are methylated faster. For example, the M.SssI target sequence shows high values for EP and ProT at the CpG site, which are not favorable for catalysis at the G position, indicating that target sites with flanking sequences reducing the EP and ProT values at the G residue are methylated more efficiently. In some cases these anticorrelations between MTase shape preferences and intrinsic DNA shape extend over several base pairs as for Opening and HelT in M.HhaI or Roll in MspI. These analyses provide novel and unique insights into the changes of intrinsic DNA conformations occurring in the MTase–DNA complexes during transition state formation.

## Conclusion

We have studied the specificities and flanking sequence preferences of six bacterial DNA-(cytosine C5)-MTases, selected to represent the entire group of these enzyme. For the structurally characterized M.HhaI and M.HaeIII, 24 enzyme mutants were generated and studied, which selectively affect defined enzyme–DNA contacts. Our study has revealed several interesting and unexpected observations that shed light on the mechanism of the DNA recognition and catalysis of this class of enzymes.

All studied bacterial MTases showed very high target motif specificities, much higher than the specificities observed for example with DNMT3A and DNMT3B [39–41]. This finding indicates that a strong selection pressure against promiscuous DNA methylation occurred in different bacterial species. Future structural and modelling studies with cognate and near-cognate substrates will be required to understand the mechanistic basis of this highly accurate sequence readout.

Our study revealed strong flanking sequence preferences of all investigated MTases, which might appear unexpected at first sight given the biological role of MTases in restriction/modification systems which is to methylate the bacterial genome at all target sites. However, flanking sequence preferences may be unavoidable, as the formation of sufficiently stable protein–DNA complexes requires an interaction of the protein with more than the 2 or 4 bp of the core recognition motif. The additional DNA interactions formed outside of the recognition motif automatically lead to DNA shape readout and flanking sequence preferences. Moreover, as restriction enzymes typically do not introduce double strand cuts into hemimethylated DNA, the time window for DNA methylation after DNA replication extends up to the next round of DNA replication. Hence, there may have been only a moderate evolutionary pressure for highly efficient and equal methylation of all target sites in every flanking context.

Most MTases displayed enzyme-specific flanking sequence preferences. This observation suggests that each enzyme has a specific and unique mode of DNA interaction and unique DNA structure in the transition state, despite their evolutionary relatedness. Only in case of M.HpaII (CCGG) and M.MspI (CCGG), similarities in the flanking sequence preferences were observed, which may reflect the closer evolutionary relationship of these enzymes.

Our data show that flanking sequence preferences change if near-cognate sites are methylated, indicating that the DNA conformation in the transition states of these methylation reactions are distinct from that of the cognate transition state. Near-cognate site methylation transition state structures may include different structural rearrangement of the DNA after base flipping as well as distinct DNA and enzyme conformations. Future structural and modelling studies with cognate and near-cognate substrates will be required to understand the structural basis of these effects.

In some cases, removal of a defined MTase–DNA contact led to local changes of the enzyme specificity and flanking sequence preferences. In other cases, long-distance effects on flanking sequence preferences were observed that extended far beyond the point of the affected enzyme–DNA contact, indicating that allosteric conformational changes of the enzyme–DNA complex are triggered by the mutations.

In several cases, we observed effects of amino acid exchanges on the DNA interacting residues on methylation activity, specificity, and flanking sequence preferences that were not explained by the enzyme–DNA structures. Based on this, we propose that the kinetically limiting step of the MTase reaction is the attack of the active site Cys residue on the flipped target base. The transition state of this reaction must lie in between double stranded DNA and the structurally resolved complexes with the target base flipped and covalently linked to the MTase. Hence, in this transition state, enzyme DNA contacts may be relevant that are not formed in the structurally resolved covalent enzyme–DNA complexes.

With M.HhaI and M.HaeIII, similar effects on flanking sequence preferences were observed with different mutations affecting different enzyme–DNA contacts. This result suggests that alternative and distinct catalytically active conformations of these enzyme-DNA complexes exist and the mutations affecting DNA contacts shift the conformational preference for one or the other in a similar way as changes in the target motif or flanking sequences do.

We observed strong, but highly enzyme specific, correlations between flanking sequence preferences and DNA shape properties with all MTases. This observation suggests that the flanking sequence dependent modulation of the DNA conformation leads to the observed MTase flanking sequence preferences. Furthermore, we derived site and DNA shape parameter specific preferences of the different enzymes providing novel insights into the transition state conformation of MTase–DNA complexes. By comparison of DNA shape preferences with the intrinsic target sequence conformations insights on the dynamic processes during transition state formation could be obtained.

In summary, our data illustrate that the DNA interaction of DNA MTases is a complex process that includes conformational changes of the enzyme and the DNA while the enzyme–DNA complex proceeds from an initial nonspecific binding state towards the transition state of the methylation reaction. Strong flanking sequence preferences were observed with all studied enzymes which are related to their influence on specific DNA conformations. Further structural, biochemical and modelling studies will be required to understand the dynamic processes of target site binding, base flipping and catalysis of this important class of enzymes at atomic resolution and find out, if the hypothesis that the transition state of the reaction precedes base flipping is transferable to other DNA MTases. The study reported here exploits Deep Enzymology, a unique experimental approach specific for DNA MTases where enzyme activity (DNA methylation) and DNA sequence of the methylation substrates can be determined together for individual product DNA molecules by bisulfite conversion coupled with DNA sequencing. Many of the insights made here may be generalizable to other enzymes interacting with DNA and to DNA-binding proteins, where similar high-throughput experiments to investigate the complex effects of DNA sequence on the biochemical activity are not yet available.

### Limitations of the study

DNA sequence preferences were only studied *in vitro*. Therefore, additional DNA-binding proteins blocking target sites, and/or protein recruiting DNA MTases to specific cellular binding sites on the bacterial DNA may modulate the effects observed here. Our study has identified several examples of strong and complex DNA sequence readout, but in many cases future structural and molecular dynamic simulation studies will be needed to enlighten the mechanistic basis of these effects. Aberrant DNA methylation may disturb proteins-DNA binding in the bacterial host organisms containing the studied MTases as part of restriction/modification systems. Unfortunately, not enough details are known about endogenous repressors and their DNA methylation sensitivity in these bacteria. Hence, future microbiological work will be needed to connect our data with specific biological processes in these bacteria and clarify the selective pressures that had acted though the evolutionary history of the studied enzymes.

## Supplementary Material

gkaf126_Supplemental_Files

## Data Availability

The raw sequence reads of all experiments and source data of the sequence specificities of all WT MTases are available at https://doi.org/10.18419/darus-4515. A compilation of all methylation experiments with all enzymes, number of sequencing reads, and methylation levels is provided in Supplementary Table S1 as a separate Excel file. Any additional information needed to reanalyze the data reported in this paper is available from the lead contact upon request.
