# Supplementary material for: Systematic analysis of specificities and flanking sequence preferences of bacterial DNA-(cytosine C5)-methyltransferases reveals mechanisms of enzyme- and sequence-specific DNA readout
Source: Nucleic Acids Res. 2025 Mar 4;53(5):gkaf126. doi: 10.1093/nar/gkaf126 (PMC11879396; doi:10.1093/nar/gkaf126)
Supplement: gkaf126_Supplemental_Files [file gkaf126_supplemental_files.zip › Bacterial MTases_suppl rev1_3.pdf]

Systematic analysis of specificities and flanking sequence preferences of bacterial DNA-(cytosine C5)-methyltransferases reveals mechanisms of enzyme and sequence specific DNA readout

Greta Sogl, Sabrina Pilling, Lukas Fischer, Jan Ludwig, Nahom Mihretu, Pavel Bashtrykov, Albert Jeltsch*

Supplementary Figures

Supplementary Figure 1: Illustration of the DNA shape parameter used in this study.

Supplementary Figure 2: Schematic overview of the Deep Enzymology analysis conducted here.

Supplementary Figure 3: Expression and purification of M.SssI, M.HaeIII WT and mutants, and M.HpaII.

Supplementary Figure 4: Additional information related to the experiments with M.SssI.

Supplementary Figure 5: Expression and purification of M.HhaI WT and mutants.

Supplementary Figure 6: Additional information related to the experiments with M.HhaI.

Supplementary Figure 7: Flanking sequence preferences of M.HhaI for methylation at GCGC and at near-cognate sites.

Supplementary Figure 8: Additional information related to the experiments with M.HaeIII.

Supplementary Figure 9: Additional information related to the experiments with M.HpaII, M.MspI and M.AluI.

Supplementary Figure 10: Additional information related to Figure 9.

Supplementary Figure 11: Compilation of the intrinsic DNA shape analyses of the target sequences for the DNA MTases investigated here.

Supplementary Figures

Supplementary Figure 1: Illustration of the DNA shape parameter used in this study. This image was taken from https://deepdnashape.usc.edu/help and modified.

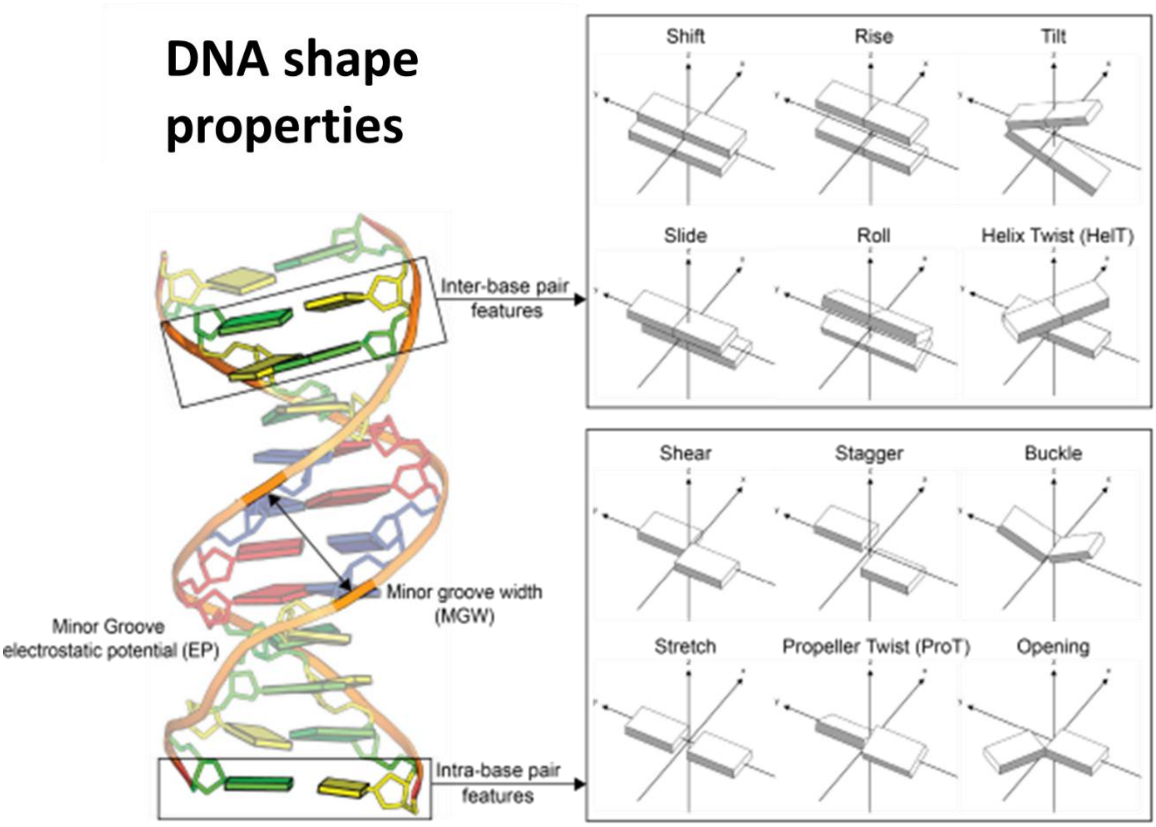

Supplementary Figure 2: Schematic overview of the Deep Enzymology analysis conducted here. A) Workflow applied to analyze DNA methylation of substrates containing random sequences, here illustrated for an example sequence containing a central CG site in an N_{10} randomized context, and an analysis of the methylation of the lower DNA strand. **B)** Enrichment/depletion analysis of bases at individual flank sites in methylated reads (here for C̲G methylation). **C)** Average methylation in specific sites, here using the GC̲GC HhaI target motif and its near-cognate sites as an example. **D)** Average methylation in NNXNN bins (here for x=C̲G). Shown as an example. In this work, most analyses were done using NNNXNNN bins.

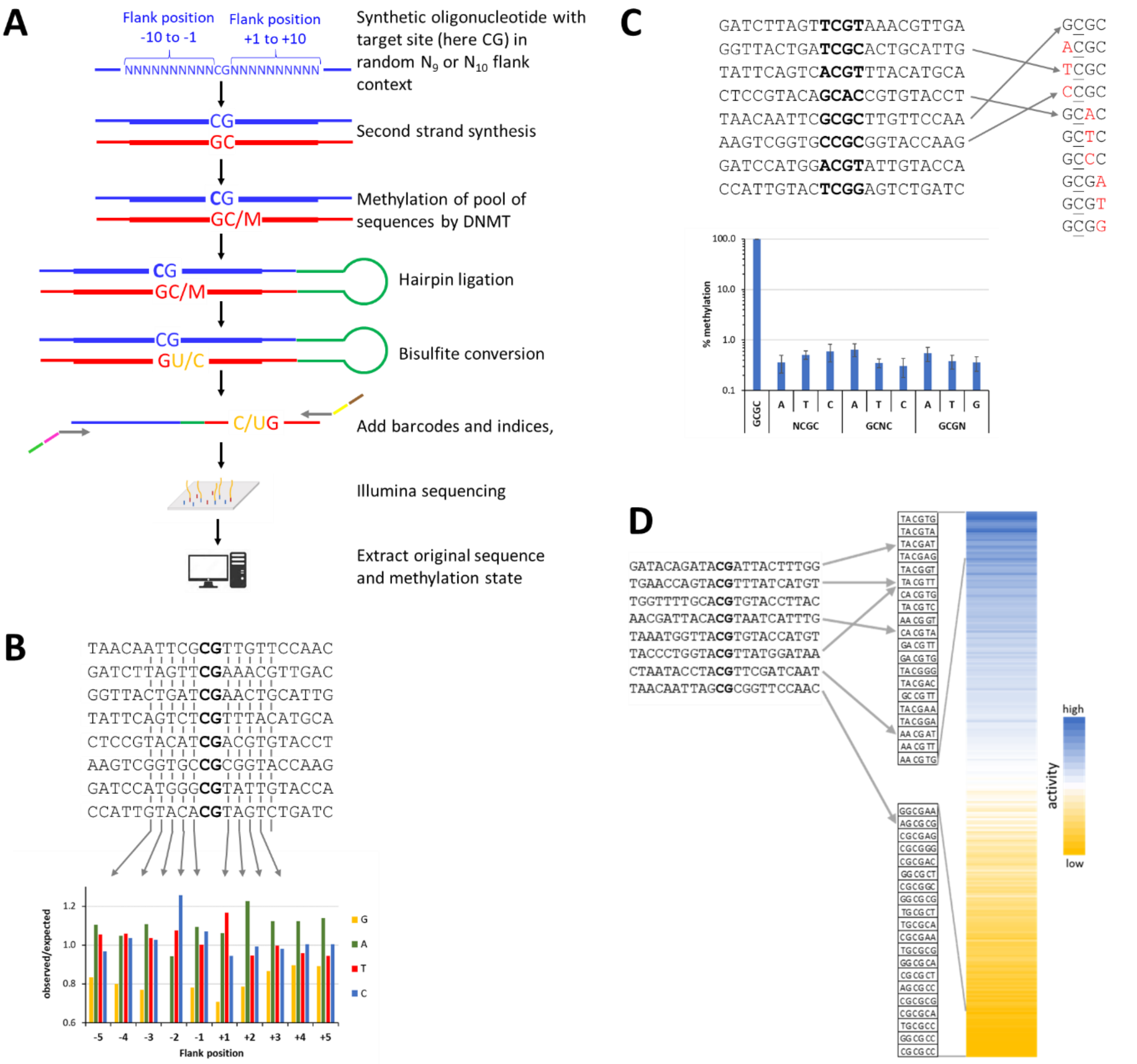

Supplementary Figure 3: Expression and purification of M.SssI (A), M.HaeIII WT and mutants (B), and M.HpaII (C). All images show Coomassie BB stained SDS-gels. The bands of expected size are labeled with asterisks. “X” refers to lanes with loading of unrelated samples. For the kinetic assays, protein concentrations were adjusted based on the intensity of the corresponding protein bands.

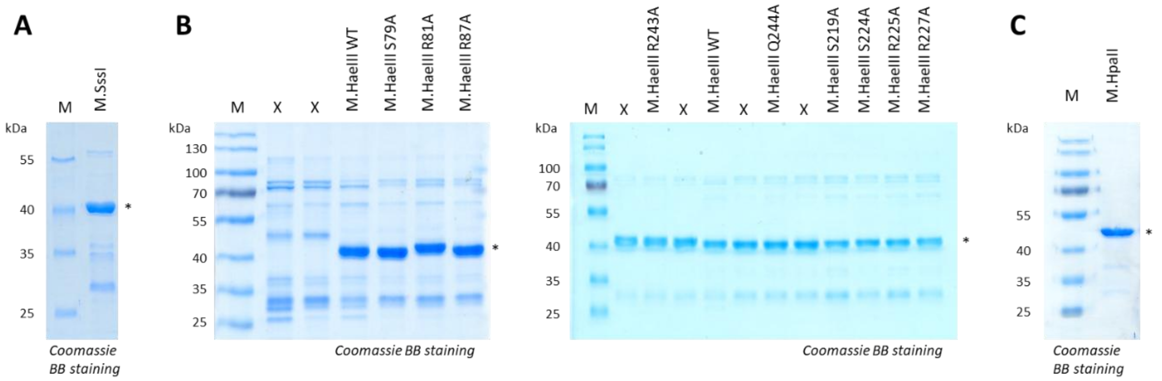

Supplementary Figure 4: Additional information related to the experiments with M.SssI. A) **E**xemplary methylation kinetics with the pool of hemimethylated CpG substrates in randomized sequence context determined using the radioactive DNA methylation assay. **B)** Correlation of methylation levels in the individual Deep Enzymology experiments with the pool of hemimethylated CpG substrates in randomized sequence context. Average methylation levels of NNCGNN bins were determined in each data set and the Pearson correlation factors of the data sets determined using Excel. Average refers to the correlation of the combined data with the individual data sets. **C)** Correlation of methylation levels in the individual Deep Enzymology experiments with the pool of CN substrates in randomized sequence context. Enrichment and depletion of bases at the -2 to +2 flank positions in CpG methylated substrate molecules were determined in each data set. Pearson correlation factors of the data sets were determined using Excel. Average refers to the correlation of the combined data with the individual data sets.

A

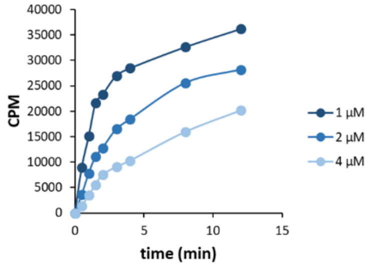

B **Hemimethylated CG substrate**

	Data set 1	Data set 2	Data set 3	Data set 4	Data set 5
Data set 1	1.0000	0.9813	0.9726	0.9804	0.9431
Data set 2	0.9813	1.0000	0.9716	0.9745	0.9405
Data set 3	0.9726	0.9716	1.0000	0.9747	0.9559
Data set 4	0.9804	0.9745	0.9747	1.0000	0.9623
Data set 5	0.9431	0.9405	0.9559	0.9623	1.0000
Average	0.9918	0.9883	0.9869	0.9927	0.9696

C **CN substrate**

	Data set 1	Data set 2	Data set 3	Data set 4	Data set 5	Data set 6
Data set 1	1.0000	0.9591	0.9747	0.9357	0.9353	0.8799
Data set 2	0.9591	1.0000	0.9710	0.9789	0.9666	0.9505
Data set 3	0.9747	0.9710	1.0000	0.9585	0.9617	0.9351
Data set 4	0.9357	0.9789	0.9585	1.0000	0.9645	0.9698
Data set 5	0.9353	0.9666	0.9617	0.9645	1.0000	0.9691
Data set 6	0.8799	0.9505	0.9351	0.9698	0.9691	1.0000
Average	0.9699	0.9902	0.9867	0.9870	0.9824	0.9658

Supplementary Figure 5: Expression and purification of M.HhaI WT (A) and mutants (B). Panel A shows an image of a Coomassie BB stained SDS-gel. Panel B shows α-His Western blot signals. The bands of expected size are labeled with asterisks. For the kinetic assays, protein concentrations were adjusted based on the intensity of the Western blot signals.

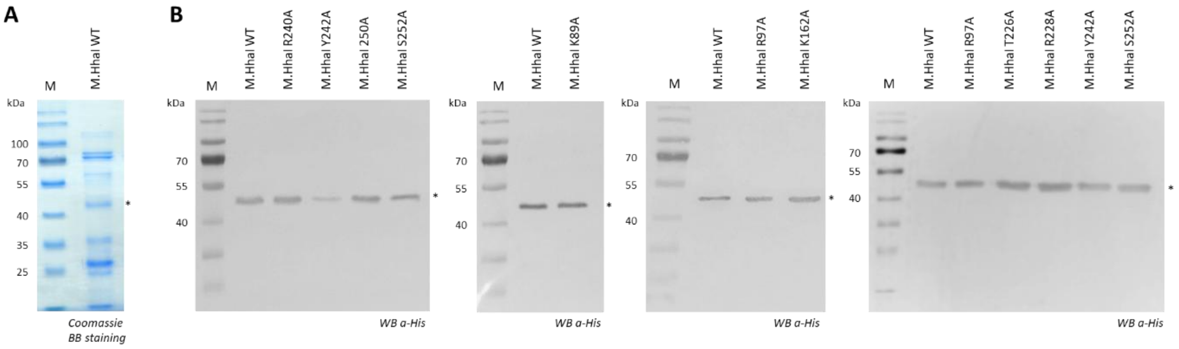

Supplementary Figure 6: Additional information related to the experiments with M.HhaI. A) Correlation of methylation levels in the individual Deep Enzymology experiments with the pool of CN substrates in randomized sequence context. Average methylation levels in GCGC and near-cognate sites were determined in each data set. Pearson correlation factors of the data sets were determined using Excel. Average refers to the correlation of the combined data with the individual data sets. **B)** Correlation of methylation levels in the individual Deep Enzymology experiments with the pool of GCGC substrates in randomized sequence context. Average methylation levels of NNGCGCNN bins were determined in each data set and the Pearson correlation factors of the data sets determined using Excel. Average refers to the correlation of the combined data with the individual data sets.

A **CN substrate**

	Data set 1	Data set 2	Data set 3	Data set 4
Data set 1	1.0000	0.9999	0.9999	1.0000
Data set 2	0.9999	1.0000	0.9999	0.9999
Data set 3	0.9999	0.9999	1.0000	0.9999
Data set 4	1.0000	0.9999	0.9999	1.0000
Average	1.0000	1.0000	0.9999	1.0000

B **GCGC substrate**

	Data set 1	Data set 2	Data set 3	Data set 4	Data set 5
Data set 1	1.0000	0.9763	0.9586	0.9409	0.9862
Data set 2	0.9763	1.0000	0.9740	0.9635	0.9666
Data set 3	0.9586	0.9740	1.0000	0.9840	0.9428
Data set 4	0.9409	0.9635	0.9840	1.0000	0.9222
Data set 5	0.9862	0.9666	0.9428	0.9222	1.0000
Average	0.9878	0.9902	0.9868	0.9766	0.9795

Supplementary Figure 7: Flanking sequence preferences of M.HhaI for methylation at GCGC and at near-cognate sites. Flanking sequence preferences were determined from the methylated near-cognate site reads after methylation of the CN substrate. They were expressed as observed/expected ratios for each base at each flanking site and color coded as heatmap from high (blue) to low (red) values.

GCGC				
Nt.	-2	-1	+1	+2
G	0.941	0.726	1.160	1.195
A	0.960	0.836	1.150	1.015
T	0.970	1.067	0.865	0.921
C	1.161	1.158	0.777	0.899

NCGC					
Site	Nt.	-2	-1	+1	+2
ACGC	G	1.15	0.62	1.07	1.03
	A	0.92	0.72	0.83	0.73
	T	0.89	0.93	0.55	1.00
	C	1.09	1.53	1.59	1.27
TCGC	G	1.24	0.45	0.50	0.93
	A	0.64	0.69	2.05	1.40
	T	0.66	1.23	0.38	0.61
	C	1.52	1.49	0.76	1.06
CCGC	G	0.87	0.55	1.15	1.70
	A	1.74	1.26	1.05	0.76
	T	0.71	1.46	1.20	0.68
	C	0.61	0.50	0.68	1.14

GCNC					
Site	Nt.	-2	-1	+1	+2
GCAC	G	1.02	1.10	1.22	0.69
	A	1.56	1.42	1.22	1.08
	T	0.54	0.94	0.84	1.07
	C	1.06	0.70	0.70	1.14
GCTC	G	0.85	0.93	0.49	0.81
	A	0.61	1.42	2.25	1.80
	T	0.88	1.07	0.26	0.73
	C	1.65	0.70	0.96	0.77
GCCC	G	0.35	1.10	0.91	0.98
	A	1.83	0.00	1.04	0.00
	T	1.42	1.35	1.54	0.89
	C	0.35	1.21	0.39	2.00

GCGN					
Site	Nt.	-2	-1	+1	+2
GCGA	G	0.63	0.37	2.00	0.98
	A	1.61	1.68	0.96	0.87
	T	0.65	0.55	1.09	0.60
	C	0.96	1.24	0.52	1.48
GCGT	G	1.20	0.00	0.86	0.94
	A	0.29	1.19	0.58	1.02
	T	2.22	0.80	1.24	1.03
	C	0.26	1.45	1.18	0.98
GCGG	G	0.00	2.25	0.00	0.00
	A	1.90	0.68	1.38	1.73
	T	0.00	0.61	1.12	1.23
	C	1.64	1.00	1.06	0.57

Supplementary Figure 8: Additional information related to the experiments with M.HaeIII. A) Correlation of methylation levels in the individual Deep Enzymology experiments with the pool of CN substrates in randomized sequence context. Average methylation levels in GGCC and near-cognate sites were determined in each data set. Pearson correlation factors of the data sets were determined using Excel. Average refers to the correlation of the combined data with the individual data sets. **B)** Correlation of methylation levels in the individual Deep Enzymology experiments with the pool of GGCC substrates in randomized sequence context. Average methylation levels of NNGCGCNN bins were determined in each data set and the Pearson correlation factors of the data sets determined using Excel. Average refers to the correlation of the combined data with the individual data sets. **C)** Specificity of M.HaeIII mutants for methylation in GGCC or near-cognate sites determined with the pool of DNA molecules containing a CN site in randomized sequence context. This image shows the near-cognate site activity of the mutants relative to their GGCC activity divided by the corresponding ratio of WT to highlight the changes compared to WT. The specificity of R225A is strongly altered and this is shown in Figure 6D.

A **CN substrate**

	Data set 1	Data set 2	Data set 3	Data set 4	Data set 5	Data set 6
Data set 1	1.0000	1.0000	0.9990	1.0000	1.0000	0.9997
Data set 2	1.0000	1.0000	0.9991	0.9999	1.0000	0.9998
Data set 3	0.9990	0.9991	1.0000	0.9990	0.9988	0.9997
Data set 4	1.0000	0.9999	0.9990	1.0000	0.9999	0.9997
Data set 5	1.0000	1.0000	0.9988	0.9999	1.0000	0.9997
Data set 6	0.9997	0.9998	0.9997	0.9997	0.9997	1.0000
Average	1.0000	1.0000	0.9993	0.9999	0.9999	0.9999

B **GGCC substrate**

	Data set 1	Data set 2	Data set 3	Data set 4	Data set 5
Data set 1	1.0000	0.8911	0.8690	0.7965	0.8176
Data set 2	0.8911	1.0000	0.8487	0.8020	0.8203
Data set 3	0.8690	0.8487	1.0000	0.7847	0.7948
Data set 4	0.7965	0.8020	0.7847	1.0000	0.7680
Data set 5	0.8176	0.8203	0.7948	0.7680	1.0000
Average	0.9626	0.9481	0.9214	0.8795	0.9025

C **M.HaeIII mutant specificity**

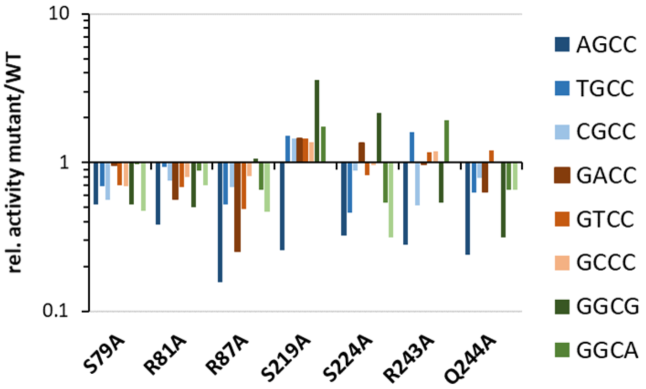

Supplementary Figure 9: Additional information related to the experiments with M.HpaII, M.MspI and M.AluI. A-C) Correlation of methylation levels in the individual Deep Enzymology experiments with the pools of target site substrates in randomized sequence context. Average target site methylation levels in all NN-X-NN bins (where X refers to the target motif of the respective enzyme) were determined in each data set and the Pearson correlation factors of the data sets determined using Excel. Average refers to the correlation of the combined data with the individual data sets. **D-F)** Correlation of methylation levels in the individual Deep Enzymology experiments with the pool of CN substrates in randomized sequence context. Average methylation levels in the respective target sites and near-cognate sites were determined in each data set. Pearson correlation factors of the data sets were determined using Excel. Average refers to the correlation of the combined data with the individual data sets.

A **M.HpaII, CCGG substrate**

	Data set 1	Data set 2	Data set 3	Data set 4
Data set 1	1.0000	0.8737	0.8382	0.8617
Data set 2	0.8737	1.0000	0.9103	0.9070
Data set 3	0.8382	0.9103	1.0000	0.9169
Data set 4	0.8617	0.9070	0.9169	1.0000
Average	0.9169	0.9647	0.9628	0.9717

B **M.MspI, CCGG substrate**

	Data set 1	Data set 2	Data set 3	Data set 4	Data set 5
Data set 1	1.0000	0.8971	0.8417	0.7729	0.7246
Data set 2	0.8971	1.0000	0.9140	0.8758	0.8273
Data set 3	0.8417	0.9140	1.0000	0.9406	0.9110
Data set 4	0.7729	0.8758	0.9406	1.0000	0.9327
Data set 5	0.7246	0.8273	0.9110	0.9327	1.0000
Average	0.8592	0.9392	0.9781	0.9721	0.9499

C **M.AluI, AGCT substrate**

	Data set 1	Data set 2	Data set 3	Data set 4	Data set 5
Data set 1	1	0.82	0.75	0.73	0.75
Data set 2	0.82	1	0.71	0.75	0.74
Data set 3	0.75	0.71	1	0.65	0.63
Data set 4	0.73	0.75	0.65	1	0.74
Data set 5	0.75	0.74	0.63	0.74	1
Average	0.93	0.91	0.83	0.87	0.86

D **M.HpaII, CN substrate**

	Data set 1	Data set 2	Data set 3	Data set 4
Data set 1	1.0000	1.0000	1.0000	1.0000
Data set 2	1.0000	1.0000	1.0000	1.0000
Data set 3	1.0000	1.0000	1.0000	1.0000
Data set 4	1.0000	1.0000	1.0000	1.0000
Average	1.0000	1.0000	1.0000	1.0000

E **M.MspI, CN substrate**

	Data set 1	Data set 2	Data set 3	Data set 4
Data set 1	1.0000	0.9999	0.9999	0.9999
Data set 2	0.9999	1.0000	0.9999	0.9999
Data set 3	0.9999	0.9999	1.0000	1.0000
Data set 4	0.9999	0.9999	1.0000	1.0000
Average	1.0000	0.9999	1.0000	1.0000

F **M.AluI, CN substrate**

	Data set 1	Data set 2	Data set 3
Data set 1	1.0000	1.0000	1.0000
Data set 2	1.0000	1.0000	1.0000
Data set 3	1.0000	1.0000	1.0000
Average	1.0000	1.0000	1.0000

Supplementary Figure 10: Additional information related to Figure 9. Average DNA methylation levels of M.HpaII, M.MspI and M.AluI substrates in all 4096 NNN-X-NNN bins.

A M.HpaII (CCGG)

Sequence	Methylation
ACC CCGG CAC	0.724
CCC CCGG GTG	0.700
CAC CCGG ATA	0.673
CCC CCGG ATG	0.667
AGA CCGG GTG	0.655
CCC CCGG GTA	0.650
CCC CCGG GTC	0.632
GCC CCGG GTA	0.629
GCC CCGG GAG	0.625
CCA CCGG GGA	0.619
ACA CCGG GTA	0.614
ATA CCGG GTA	0.613
CTA CCGG GGG	0.611
CCC CCGG TTG	0.610
TCA CCGG GTG	0.600
TCT CCGG GTG	0.600
GCC CCGG GTG	0.600
GTA CCGG ATA	0.598
TCC CCGG GTA	0.596
TAC CCGG TAC	0.595

Sequence	Methylation
AGG CCGG CAG	0.045
TGG CCGG CCC	0.042
GGT CCGG CTC	0.040
GGG CCGG CTG	0.040
CGA CCGG CCT	0.040
GAG CCGG TTG	0.037
GGG CCGG TCG	0.033
AAA CCGG CCG	0.029
AGC CCGG CCC	0.000
TAG CCGG CCC	0.000
GAG CCGG CGG	0.000
GAG CCGG CCC	0.000
GGA CCGG AGG	0.000
GGA CCGG CCC	0.000
GGG CCGG AAA	0.000
GGG CCGG CCA	0.000
GGG CCGG CCT	0.000
GGG CCGG CCG	0.000
GCC CCGG CCC	0.000
CGG CCGG CGC	0.000

B M.MspI (CCGG)

Sequence	Methylation
AAC CCGG AAT	0.661
CCC CCGG GAA	0.655
TAC CCGG TCA	0.651
CAC CCGG ACA	0.647
CAT CCGG AAT	0.645
TAC CCGG AAT	0.643
CCC CCGG AAT	0.615
TAC CCGG ACG	0.606
CTG CCGG GCA	0.606
CCC CCGG GTA	0.605
CCT CCGG AAT	0.598
CAC CCGG GAT	0.593
TAC CCGG AAC	0.590
TAC CCGG TAC	0.588
CAC CCGG TAT	0.586
TAT CCGG GAT	0.586
CCC CCGG TAC	0.576
CCA CCGG GCA	0.576
ACC CCGG AAT	0.573
TAC CCGG GAT	0.570

Sequence	Methylation
GGG CCGG GGG	0.033
GGG CCGG CAA	0.033
CGG CCGG CAT	0.032
GGG CCGG CAG	0.032
CGG CCGG CAA	0.032
GGG CCGG TCC	0.030
AGG CCGG CAC	0.030
GGG CCGG AAA	0.027
CGG CCGG CGT	0.027
CTG CCGG CGC	0.027
GGG CCGG CGC	0.026
GGG CCGG AGA	0.025
TGG CCGG CGT	0.024
GGG CCGG CGT	0.023
GGG CCGG CTC	0.020
AGG CCGG AGC	0.015
CGG CCGG AGG	0.015
AGG CCGG GTC	0.000
CGG CCGG AGT	0.000
CGG CCGG CGC	0.000

C M.AluI (AGCT)

Sequence	Methylation
AAG AGCT CAC	1.000
AGA AGCT TCT	1.000
AGA AGCT GCT	1.000
AGA AGCT CAA	1.000
AGA AGCT CTT	1.000
GGA AGCT GCT	1.000
GGA AGCT CCT	1.000
GGG AGCT CTT	1.000
GGG AGCT CCT	1.000
ATA AGCT CTC	0.962
AGT AGCT CTC	0.962
AAG AGCT ATG	0.957
AAG AGCT CTT	0.952
ACC AGCT CCA	0.952
AGA AGCT AGA	0.944
AAC AGCT CTG	0.943
AAG AGCT GCG	0.941
ATG AGCT GCT	0.941
GAG AGCT GCG	0.935
TAG AGCT GCT	0.933

Sequence	Methylation
TAC AGCT GAC	0.357
CAT AGCT GTC	0.357
GGG AGCT TAA	0.350
CTA AGCT GAG	0.341
AAA AGCT GAC	0.333
AAC AGCT GAG	0.333
GAA AGCT CCT	0.333
GGG AGCT ACC	0.333
CTC AGCT GAG	0.333
GTC AGCT GTC	0.329
GAC AGCT GTT	0.323
GAC AGCT GAG	0.323
GTC AGCT TTC	0.321
CTA AGCT TAG	0.320
GAA AGCT GAG	0.316
CTA AGCT TAA	0.308
GTC AGCT TAG	0.305
GCC AGCT GGT	0.304
GCA AGCT GAG	0.300
CTC AGCT GGC	0.286

Supplementary Figure 11: Compilation of the intrinsic DNA shape analyses of the target sequences for the DNA MTases investigated here. Values were obtained by averaging of all N_4XN_4 profiles for each target sequence (X) predicted using the Deep DNAshape server (https://deepdnashape.usc.edu/). Numbers are indicated in the corresponding units: Å for MGW, Shear, Stretch, Stagger, Rise, Shift and Slide; degree for ProT, Opening, Roll, and Tilt; mV for EP.

M.SssI

Property	Position									
	-4	-3	-2	-1	C	G	+1	+2	+3	+4
MWG	5.17	5.18	5.23	5.20	5.28	5.28	5.20	5.23	5.18	5.17
EP	-6.13	-6.11	-6.07	-5.94	-4.37	-4.37	-5.94	-6.07	-6.11	-6.13
Shear	0.00	0.00	0.00	0.01	0.26	-0.26	-0.01	0.00	0.00	0.00
Stretch	-0.03	-0.03	-0.03	-0.03	-0.03	-0.03	-0.03	-0.03	-0.03	-0.03
Stagger	-0.03	-0.01	0.00	0.02	0.00	0.00	0.02	0.00	-0.01	-0.03
Buckle	0.13	-0.46	-0.02	1.73	2.21	-2.21	-1.73	0.02	0.46	-0.13
ProT	-7.09	-6.79	-6.69	-6.30	-5.64	-5.64	-6.30	-6.69	-6.79	-7.09
Opening	-0.13	-0.25	-0.28	-0.23	0.45	0.45	-0.23	-0.28	-0.25	-0.13

Property	Position								
	-4/3	-3/2	-2/1	-1/C	C/G	G/+1	+1/2	+2/3	+3/4
Shift	0.00	-0.01	0.01	0.10	0.00	-0.10	-0.01	0.01	0.00
Slide	-1.53	-1.51	-1.48	-1.55	-1.62	-1.55	-1.48	-1.51	-1.53
Rise	3.34	3.33	3.29	3.35	3.42	3.35	3.29	3.33	3.34
Tilt	-0.02	-0.03	-0.07	-0.16	0.00	0.16	0.07	0.03	0.02
Roll	-0.59	-0.57	-0.72	-1.59	3.68	-1.59	-0.72	-0.57	-0.59
HelT	34.35	34.28	34.27	34.95	33.08	34.95	34.27	34.28	34.35

M.HhaI

Property	Position											
	-4	-3	-2	-1	G	C	G	C	+1	+2	+3	+4
MWG	5.17	5.19	5.24	5.39	5.38	5.54	5.54	5.38	5.39	5.24	5.19	5.17
EP	-6.12	-6.09	-6.05	-5.73	-4.56	-4.10	-4.10	-4.56	-5.73	-6.05	-6.09	-6.12
Shear	0.00	0.00	0.00	0.00	-0.26	0.25	-0.25	0.26	0.00	0.00	0.00	0.00
Stretch	-0.03	-0.03	-0.03	-0.03	-0.03	-0.03	-0.03	-0.03	-0.03	-0.03	-0.03	-0.03
Stagger	-0.03	-0.01	-0.01	-0.01	0.09	0.06	0.06	0.09	-0.01	-0.01	-0.01	-0.03
Buckle	0.11	-0.47	-0.15	0.64	0.60	0.04	-0.04	-0.60	-0.64	0.15	0.47	-0.11
ProT	-7.10	-6.79	-6.75	-6.15	-3.31	-3.94	-3.94	-3.31	-6.15	-6.75	-6.79	-7.10
Opening	-0.13	-0.25	-0.29	-0.24	0.27	0.37	0.37	0.27	-0.24	-0.29	-0.25	-0.13

Property	Position										
	-4/3	-3/2	-2/1	-1/G	G/C	C/G	G/C	C/+1	+1/2	+2/3	+3/4
Shift	0.00	-0.01	0.02	0.16	-0.01	0.00	0.01	-0.16	-0.02	0.01	0.00
Slide	-1.53	-1.52	-1.52	-1.59	-1.56	-1.59	-1.56	-1.59	-1.52	-1.52	-1.53
Rise	3.34	3.33	3.31	3.33	3.39	3.32	3.39	3.33	3.31	3.33	3.34
Tilt	-0.02	-0.01	0.03	-0.75	0.38	0.00	-0.38	0.75	-0.03	0.01	0.02
Roll	-0.50	-0.49	-0.62	0.98	-1.41	2.79	-1.41	0.98	-0.62	-0.49	-0.50
HelT	34.33	34.31	34.04	33.24	36.11	32.48	36.11	33.24	34.04	34.31	34.33

M.HaeIII

Property	Position											
	-4	-3	-2	-1	G	G	C	C	+1	+2	+3	+4
MWG	5.17	5.20	5.28	5.31	5.17	4.90	4.90	5.17	5.31	5.28	5.20	5.17
EP	-6.12	-6.08	-6.00	-5.73	-3.98	-4.10	-4.10	-3.98	-5.73	-6.00	-6.08	-6.12
Shear	0.00	0.00	0.00	0.00	-0.26	-0.28	0.28	0.26	0.00	0.00	0.00	0.00
Stretch	-0.03	-0.03	-0.03	-0.03	-0.04	-0.04	-0.04	-0.04	-0.03	-0.03	-0.03	-0.03
Stagger	-0.03	-0.01	-0.01	-0.01	0.08	0.04	0.04	0.08	-0.01	-0.01	-0.01	-0.03
Buckle	0.11	-0.49	0.23	1.80	1.03	0.55	-0.55	-1.03	-1.80	-0.23	0.49	-0.11
ProT	-7.10	-6.77	-6.45	-5.63	-2.61	-2.59	-2.59	-2.61	-5.63	-6.45	-6.77	-7.10
Opening	-0.13	-0.25	-0.27	-0.13	0.25	0.36	0.36	0.25	-0.13	-0.27	-0.25	-0.13

Property	Position										
	-4/3	-3/2	-2/1	-1/G	G/G	C/G	C/C	C/+1	+1/2	+2/3	+3/4
Shift	0.00	0.00	0.03	0.18	0.10	0.00	-0.10	-0.18	-0.03	0.00	0.00
Slide	-1.53	-1.52	-1.53	-1.66	-1.79	-1.62	-1.79	-1.66	-1.53	-1.52	-1.53
Rise	3.34	3.33	3.29	3.35	3.37	3.41	3.37	3.35	3.29	3.33	3.34
Tilt	-0.02	-0.02	0.02	-0.94	0.69	0.00	-0.69	0.94	-0.02	0.02	0.02
Roll	-0.50	-0.49	-0.82	0.42	-1.79	-2.31	-1.79	0.42	-0.82	-0.49	-0.50
HelT	34.33	34.26	33.84	33.20	33.51	36.72	33.51	33.20	33.84	34.26	34.33

M.HpaII
M.MspI

Property	Position											
	-4	-3	-2	-1	C	C	G	C	+1	+2	+3	+4
MWG	5.17	5.17	5.17	5.05	5.01	5.30	5.30	5.01	5.05	5.17	5.17	5.17
EP	-6.13	-6.13	-6.10	-6.00	-4.55	-4.06	-4.06	-4.55	-6.00	-6.10	-6.13	-6.13
Shear	0.00	0.00	0.00	0.01	0.27	0.25	-0.25	-0.27	-0.01	0.00	0.00	0.00
Stretch	-0.03	-0.03	-0.03	-0.03	-0.04	-0.04	-0.04	-0.04	-0.03	-0.03	-0.03	-0.03
Stagger	-0.03	-0.01	-0.01	0.00	0.02	0.04	0.04	0.02	0.00	-0.01	-0.01	-0.03
Buckle	0.13	-0.36	0.12	1.36	1.01	0.39	-0.39	-1.01	-1.36	-0.12	0.36	-0.13
ProT	-7.09	-6.76	-6.48	-5.91	-4.41	-3.72	-3.72	-4.41	-5.91	-6.48	-6.76	-7.09
Opening	-0.13	-0.24	-0.23	-0.13	0.31	0.30	0.30	0.31	-0.13	-0.23	-0.24	-0.13

Property	Position										
	-4/3	-3/2	-2/1	-1/C	C/C	C/G	G/G	G/+1	+1/2	+2/3	+3/4
Shift	0.00	0.00	0.03	0.09	-0.09	0.00	0.09	-0.09	-0.03	0.00	0.00
Slide	-1.53	-1.52	-1.51	-1.60	-1.74	-1.65	-1.74	-1.60	-1.51	-1.52	-1.53
Rise	3.34	3.33	3.30	3.37	3.37	3.34	3.37	3.37	3.30	3.33	3.34
Tilt	-0.02	-0.02	0.02	-0.54	-0.37	0.00	0.37	0.54	-0.02	0.02	0.02
Roll	-0.62	-0.75	-1.06	-1.78	-1.07	1.90	-1.07	-1.78	-1.06	-0.75	-0.62
HelT	34.35	34.27	34.18	35.33	33.59	32.59	33.59	35.33	34.18	34.27	34.35

M.AluI

Property	Position											
	-4	-3	-2	-1	A	G	C	T	+1	+2	+3	+4
MWG	5.18	5.22	5.29	5.38	5.24	4.70	4.70	5.24	5.38	5.29	5.22	5.18
EP	-6.11	-6.07	-6.07	-6.15	-7.33	-5.01	-5.01	-7.33	-6.15	-6.07	-6.07	-6.11
Shear	0.00	0.00	0.00	0.00	0.10	-0.24	0.24	-0.10	0.00	0.00	0.00	0.00
Stretch	-0.03	-0.03	-0.03	-0.03	-0.02	-0.02	-0.02	-0.02	-0.03	-0.03	-0.03	-0.03
Stagger	-0.03	-0.01	0.00	-0.05	-0.02	0.13	0.13	-0.02	-0.05	0.00	-0.01	-0.03
Buckle	0.06	-0.52	0.17	0.57	2.32	2.63	-2.63	-2.32	-0.57	-0.17	0.52	-0.06
ProT	-7.11	-6.85	-6.68	-7.45	-7.23	-1.48	-1.48	-7.23	-7.45	-6.68	-6.85	-7.11
Opening	-0.13	-0.28	-0.33	-0.33	-0.63	0.32	0.32	-0.63	-0.33	-0.33	-0.28	-0.13

Property	Position										
	-4/3	-3/2	-2/1	-1/A	A/G	G/C	C/T	T/+1	+1/2	+2/3	+3/4
Shift	-0.01	-0.01	-0.02	-0.08	0.27	0.00	-0.27	0.08	0.02	0.01	0.01
Slide	-1.53	-1.51	-1.50	-1.42	-1.78	-1.76	-1.78	-1.42	-1.50	-1.51	-1.53
Rise	3.34	3.32	3.34	3.27	3.34	3.52	3.34	3.27	3.34	3.32	3.34
Tilt	-0.02	-0.09	0.28	-0.20	-1.76	0.00	1.76	0.20	-0.28	0.09	0.02
Roll	-0.50	-0.52	-0.14	0.87	-2.70	-3.30	-2.70	0.87	-0.14	-0.52	-0.50
HelT	34.34	34.31	34.20	34.95	31.50	37.77	31.50	34.95	34.20	34.31	34.34
